# Coexisting traditional and biomedical healthcare systems: a mixed-methods analysis of community health workers and traditional birth attendants' contributions to perinatal health behaviors in rural India

**DOI:** 10.3389/frhs.2025.1623426

**Published:** 2025-12-18

**Authors:** Faiz A. Hashmi, Oskar Burger, Cristine H. Legare

**Affiliations:** 1 Center for Applied Cognitive Science, The University of Texas at Austin, Austin, TX, United States; 2 OMNI Institute, Denver, CO, United States

**Keywords:** accredited social health activist (ASHA), cultural practices and beliefs, health behavior change, maternal and child health, mixed methods research, therapeutic pluralism, traditional birth attendant (Dai), India

## Abstract

In many rural communities, traditional and biomedical health systems operate side by side, yet the comparative roles of traditional birth attendants and community health workers in perinatal care remain poorly understood. This study examines the variations in the influence of Accredited Social Health Activists (ASHAs) and traditional birth attendants (locally known as Dais) on maternal and newborn health behaviors in rural Bihar, India. We employed a mixed-methods design. Qualitative data included 40 focus group discussions, 50 key informant interviews, and six weeks of focused ethnographic observation of both ASHAs and Dais guided by rapid ethnography principles. Quantitative data were collected through a multi-stage cluster random survey of 1,166 recent mothers and 400 ASHAs, designed to ensure representation across Bihar's major linguistic regions. Logistic regression with backward selection (validated through sensitivity analyses and alternate specifications) estimated the influence of each provider on perinatal behaviors, with multicollinearity assessed using variance inflation factors. Findings revealed distinct temporal and functional roles: ASHAs were most active during pregnancy and labor, significantly increasing the odds of antenatal visits and institutional delivery, while Dais exerted greater influence postpartum, promoting traditional practices such as newborn massage. Synergistic effects emerged in breastfeeding initiation when both providers were involved, while conflicting guidance appeared in cord care. Families often created hybrid care models that blended biomedical recommendations with ritual practices. Overall, the coexistence of ASHAs and Dais suggests complementarity rather than simple competition, though patterns varied across settings. The study focuses on behaviors rather than health outcomes, and we acknowledge that this scope, along with ethical considerations of working with overlapping provider systems, shapes interpretation. Tailored strategies that foster respectful collaboration—such as joint training and coordinated outreach—may improve the uptake and cultural acceptability of maternal and newborn health programs in rural contexts.

## 1 Background

The coexistence of traditional and biomedical healthcare systems is universal (1, 2). Biomedical systems, often referred to as conventional or Western medicine, are grounded in scientific principles and evidence-based practices focused on disease diagnosis and treatment, while traditional health systems encompass culturally specific practices that integrate physical, spiritual, and social dimensions of well-being (3–5). As the world strives to achieve ambitious maternal and child health targets outlined in the Sustainable Development Goals, understanding how these parallel systems interact and influence health outcomes has become increasingly crucial (6, 7).

The interplay between biomedical and traditional health systems becomes particularly noticeable in reproductive, maternal, and child health domains, where culturally rooted practices frequently intersect with modern medical protocols (8, 9). During the perinatal period (time from conception to one month after childbirth), families commonly draw on both, weaving traditional health practices and rituals with standardized medical protocols to safeguard mothers and infants (for instance, women may follow dietary restrictions prescribed by elders—such as avoiding “hot” foods—while also taking iron and folic acid tablets from health workers) (10–13). Healthcare decisions during pregnancy and childbirth are deeply embedded in cultural and religious beliefs, such as observing fasting rituals on auspicious days to have a healthy child (14). Even when biomedical care is available and accessible, communities often perceive many traditional health practices as aligned with their values and needs as well (15).

The progression from traditional to biomedical health practices in rural India is not linear; rather, it presents a layered landscape where traditional wisdom and biomedical knowledge are actively integrated (16, 17). For example, in Bihar, families might choose hospital delivery while maintaining culturally significant religious rituals believed to ward off supernatural threats, such as burning wood at home entrances or bringing iron objects (11). Similarly, while medical professionals advocate immediate breastfeeding, many mothers prioritize religious rites, such as waiting for spiritual blessings, before following biomedical recommendations. These practices reflect the belief that spiritual protection is necessary alongside biomedical care, illustrating an integration rather than a substitution of healthcare approaches. Rather than following a linear path, biomedical recommendations and spiritual or ritualistic beliefs merge in complex ways, shaping health-seeking behaviors as families navigate both systems simultaneously (18, 19).

The perinatal health landscape is often a complex interaction between historically rooted traditions and modern biomedical interventions (20, 21). This is particularly evident in rural India, where Traditional Birth Attendants (locally known as Dais) and Accredited Social Health Activists (ASHAs) act as crucial, yet distinct, health influencers (22). Their coexistence embodies the dynamic between traditional health systems and biomedical protocols, directly shaping community care-seeking behaviors and maternal health outcomes (19, 23, 24).

Dais have been central figures during pregnancy and childbirth in India for centuries. As representatives of traditional healthcare systems, Dais embody centuries of accumulated knowledge and traditional health practices in perinatal care (25, 26). Dais are trusted community members, cultural authorities, and keepers of traditional knowledge passed down through generations (27, 28). Their influence stems from their skills in managing childbirth, and their deep understanding of local customs, spiritual beliefs, and social dynamics shape perinatal health behaviors (15, 18). Their influence extends beyond the aspects of delivery, including psychological, social, and spiritual support, functioning as bridges between traditional health practices and perinatal behaviors (29). However, with the modernization of maternal and child health services through government programs, there is a perception that Dais are becoming obsolete due to transformations in service delivery and the growing role of community health workers (30).

ASHA program represents one of India's most ambitious efforts to modernize rural healthcare (31). These community health workers serve as critical links between villages and the formal healthcare system, striving to increase institutional deliveries, improve antenatal care uptake, and promote evidence-based practices (32, 33). Their effectiveness in raising perinatal health indicators—especially institutional deliveries and vaccination rates—is well-documented (34, 35). Yet, their success frequently depends on their capacity to facilitate and articulate contemporary healthcare protocols within the framework of sociocultural acceptance (36, 37).

Under the National Health Mission, India officially recognizes ASHAs and emphasizes hospital births, but largely sidelines Dais, despite many rural communities continuing to consult Dais (38). Performance-linked incentives push ASHAs toward institutional deliveries, often placing them in de facto competition with Dais and keeping collaboration *ad hoc* in remote areas (39). The distinction between ASHAs and Dais extends beyond their healthcare philosophies to their training and legitimacy. ASHAs undergo structured government training programs, receive stipends for specific services, and operate within defined job descriptions as part of India's National Rural Health Mission (40, 41). In contrast, Dais typically acquire their knowledge through apprenticeship models, often inheriting their practice through maternal lineages, with their authority derived from community trust and cultural legitimacy rather than formal certification (42). Given the presence of both Dais and ASHAs, examining the broader perinatal health outcomes in rural India becomes imperative.

While India has improved perinatal health outcomes, specific key indicators have plateaued in many rural regions (43, 44). This stagnation reflects gaps in healthcare infrastructure and the socio-cultural factors influencing the adoption of biomedical recommendations (45, 46). Such factors highlight the reliance on traditional and biomedical health systems, underscoring the need to explore how each system contributes to perinatal healthcare progress or sometimes hinders it. At the same time, despite extensive research on both ASHAs and Dais independently, a critical gap remains in our understanding of how these two groups of providers interact and collectively influence perinatal health behaviors (47–49). Studies often focus on Dais' cultural authority or the institutional impact of ASHAs, but few investigate how communities negotiate the presence of both. Rural families commonly maintain relationships with both providers, basing healthcare decisions on trust, accessibility, cultural alignment, and perceived effectiveness (50). Consequently, bridging this gap in knowledge is vital for more comprehensive and culturally sensitive healthcare interventions.

The dual presence of ASHAs and Dais offers a unique opportunity to investigate the interplay between traditional and biomedical health systems, shedding light on their comparative roles in shaping perinatal behaviors and practices (29, 34). Understanding the interactions between ASHAs and Dais—synergistic or antagonistic—is critical for developing integrated healthcare strategies that leverage traditional and biomedical health systems (51). The coexistence of these two groups of health practitioners provides valuable insights into how communities negotiate between traditional wisdom and modern medical knowledge and how this negotiation influences perinatal healthcare decisions (52). Moreover, examining these dynamics can reveal potential pathways for integrating traditional and biomedical approaches to enhance rather than diminish the effectiveness of perinatal healthcare delivery (53).

Our research employs an exploratory sequential mixed methods approach to illuminate this complex dynamic by comparing the roles of ASHAs and Dais in influencing perinatal behaviors within rural Indian communities. Specifically, we address three questions: (1) Are there variations in how ASHAs and Dais influence perinatal healthcare decisions? (2) How do they exert their authority or guidance? (3) How do communities reconcile potentially conflicting recommendations from these two groups? By answering these questions, we offer actionable insights into how leveraging both traditional and biomedical health systems can improve maternal and infant well-being in a culturally resonant and effective manner.

## 2 Methods

### 2.1 Study setting and design

This study employed a mixed-methods approach as part of Project RISE to investigate the comparative roles and influences of Accredited Social Health Activists (ASHAs) and traditional birth attendants (Dais) on perinatal health behaviors in rural Bihar, India. Bihar represents an ideal setting for this research as India's most rural and economically disadvantaged state, where traditional and biomedical healthcare systems coexist in complex ways that shape maternal and child health outcomes. Our research design integrated qualitative, ethnographic, and quantitative components to triangulate findings across multiple data sources.

For this study, we define the perinatal period as the time from conception to one month postpartum. This definition is broader than the WHO's clinical definition (22 weeks of gestation to 7 days after birth) but captures the full spectrum of traditional and biomedical care practices during this critical period, including early pregnancy practices and extended postpartum cultural rituals that influence health outcomes.

The study employed an exploratory sequential mixed-methods design, in which the qualitative phase (focus groups, key informant interviews, and ethnographic observations) informed the development and refinement of the quantitative survey instruments. Ethnographic methods followed focused ethnography principles, emphasizing intensive observation over shorter time periods to capture healthcare provider practices and community dynamics.

### 2.2 Qualitative methods

#### 2.2.1 Focus group discussions (FGDs)

##### 2.2.1.1 Participant recruitment

For focus group discussions, we purposively selected two distinct participant groups in collaboration with local health officials to capture intergenerational perspectives on healthcare provider preferences and perinatal health practices. Participants were recruited from the catchment areas of Anganwadi centers (AWCs) based on specific inclusion criteria: younger mothers must have given birth within the past two years, while older mothers must have a married daughter or daughter-in-law. Within these groups, we ensured variation across caste categories, education levels, and economic status to capture diverse experiences with healthcare providers.

##### 2.2.1.2 Data collection

Focus group discussions were conducted across 21 villages in Nalanda and Samastipur districts during January 14–25, 2019. These districts represent Bihar's sociocultural diversity: Nalanda in South Bihar's alluvial plains with Magahi-speaking populations, and Samastipur in North Bihar plains with Maithili-speaking communities. While not representative of all 38 districts, they capture key variations in agricultural systems, linguistic communities, and urban proximity affecting healthcare access patterns and provider preferences.

Forty FGDs were conducted, equally split between younger and older mothers, with participants typically segregated by religion to facilitate comparative analysis and encourage open discussion of sensitive health topics. The groups ranged in size from 4 to 7 participants, with a median of 5 participants per discussion.

Data collection was conducted by trained field researchers from Project Concern International (PCI), including 6 female research assistants with 4–10 years of experience in rural health research in Bihar. The research team included bilingual speakers of Hindi, Maithili, Magahi, and Bhojpuri, with prior experience working with similar rural populations. All research assistants received 40 h of training on qualitative data collection methods, cultural sensitivity, and ethical considerations specific to maternal health research.

Each FGD was facilitated by one primary moderator with one note-taker present. Semi-structured discussion guides explored participants' health-related beliefs and practices throughout the perinatal period, including preferences for different healthcare providers, experiences with biomedical and traditional care, perceived effectiveness of different providers, and decision-making processes for healthcare choices. Discussions examined perceived health threats, preventive practices, and the reasoning behind specific health behaviors and provider selection.

All sessions were audio-recorded with basic demographic information collected from participants. Detailed field notes documented non-verbal cues, group dynamics, and contextual observations. Researchers employed follow-up questions to resolve ambiguities while maintaining natural conversational flow.

Researchers had no prior relationships with study participants. To minimize social desirability bias, research assistants emphasized confidentiality, used neutral language when discussing sensitive topics, conducted interviews in private settings when possible, and were rotated across different villages to prevent familiarity effects. Participants were assured that their responses would not be shared with local health workers or family members.

Data collection continued until thematic saturation was observed during preliminary analysis, assessed after every five interviews, with no new themes emerging from the final eight focus group discussions.

Our analytical approach involved rapid descriptive coding of each practice or belief statement, followed by inductive thematic grouping to identify patterns across the data. This two-stage process allowed initial categorization of discrete practices while remaining open to emergent themes that cut across predetermined categories. To enhance analytical rigor and identify dominant narratives, we conducted frequency tallies by speaker type and district, integrating quantitative counting with qualitative interpretation. Two independent coders performed peer debriefing throughout the analysis, with discrepancies resolved through consensus discussion to ensure coding reliability and interpretive validity.

#### 2.2.2 Key informant interviews (KIIs)

##### 2.2.2.1 Participant recruitment

Key Informant Interviews were conducted concurrently with FGDs in the same 21 villages during January 14–25, 2019. We employed a combination of snowball and convenience sampling to recruit community influencers who shape maternal health decision-making and represent different perspectives on perinatal care. Key informants encompassed five categories: Community Health Workers including ASHAs and AWWs identified through local health centers; Traditional Birth Attendants identified through community referrals; Registered Medical Practitioners from both government and private sectors; and religious leaders including Hindu Pandits and Muslim Maulanas recruited through local religious institutions.

##### 2.2.2.2 Data collection

We conducted fifty key informant interviews across five stakeholder categories representing the maternal health ecosystem. Community health workers comprised the largest group with 12 ASHAs and 11 Anganwadi Workers. Traditional health providers included 10 Dais, medical practitioners consisted of 6 government and private doctors, and religious leaders included 5 Hindu Pandits and 6 Muslim Maulanas, providing crucial insights into faith-based healthcare preferences and cultural practices affecting provider selection.

Interview guides were designed to parallel FGD prompts, enabling systematic comparison across data collection methods and triangulation of findings. Semi-structured interview guides covered healthcare provider knowledge and practices, interaction with other providers, community trust and authority, barriers and facilitators to healthcare delivery, and perspectives on traditional vs. biomedical care.

Interviews lasted approximately 45–60 min and were conducted by single interviewers. All interviews were recorded, transcribed verbatim, and translated from Hindi and local dialects by bilingual research assistants with expertise in health terminology. Back-translation was performed by independent translators to ensure accuracy.

Analysis combined deductive and inductive approaches, with initial rapid coding using predetermined domains (provider roles, community perceptions, healthcare delivery, traditional-biomedical interactions) while remaining open to emergent themes. Domain grouping organized coded segments into higher-level categories to reveal patterns across stakeholder groups. Frequency counts identified recurring themes by informant type, quantifying which issues were most salient for different groups while preserving context. Strategic quote selection balanced representative responses with unique insights that revealed exceptional cases.

#### 2.2.3 Ethnographic component

##### 2.2.3.1 Participant recruitment

For the ethnographic fieldwork, ASHAs were selected using criterion-based sampling focused on diversity in years of service and village settings. Selection criteria included active service in their communities and variation in experience levels. Three Dais were also selected for observation during home visits and community interactions, identified through community recommendations and willingness to participate. Participating households were selected based on having either a pregnant woman or a lactating mother with an infant under six months, ensuring opportunities to observe healthcare provider interactions during critical perinatal periods.

##### 2.2.3.2 Data collection

We conducted focused ethnographic fieldwork in the Samastipur district from February to March 2019. This six-week intensive period was strategically timed to coincide with key maternal health events and provider activities. Ethnographic observations totaled 45 days across 4 villages, with each of the 8 selected ASHAs shadowed for 2–3 consecutive days.

The ethnographic component employed focused ethnographic methods following Knoblauch's rapid ethnography framework, emphasizing intensive observation over shorter time periods. A cultural anthropologist and two field assistants employed various ethnographic methods, including in-depth interviews, specialized FGDs, ASHA shadowing, and participant observation.

The field team shadowed 8 ASHAs across four blocks, observing them in multiple contexts: home visits during Home-Based Newborn Care, community health meetings at Anganwadi centers, immunization sessions, family planning days at Primary Health Centers, and informal village interactions. The investigation included extensive field observations of ASHAs' daily activities in their homes and community settings. Researchers also observed a five-day residential ASHA training session and monthly meetings at panchayat-level health subcenters and block-level Primary Health Centers.

Observations documented healthcare provider interactions, advice provided to families, community responses to different providers, conflicts or collaboration between traditional and biomedical providers, and family decision-making processes regarding healthcare choices. These observations were supplemented by opportunistic informal interviews with an ASHA trainer, two Auxiliary Nurse Midwives, traditional birth attendants, and multiple family members present during visits.

Field notes were recorded using structured templates covering provider behaviors, community responses, and contextual factors influencing healthcare delivery. All observations and informal conversations were documented through detailed field notes written within 24 h of each session.

Data were recorded as detailed narrative field notes and thematically coded according to three primary domains: provider authority and influence mechanisms, community perceptions and trust patterns, and healthcare system integration or conflict. This ethnographic component provided rich contextual insights into how different healthcare providers operate within community dynamics and cultural constraints.

### 2.3 Quantitative survey

#### 2.3.1 Participant recruitment

For the quantitative survey, we implemented a multi-stage cluster random sampling across Bihar's three major linguistic regions: Maithili (Samastipur and Purnia districts), Magahi (Gaya district), and Bhojpuri (West Champaran district). This sampling approach ensured representation from Bihar's major dialectical regions while upholding methodological integrity.

In the first stage, two blocks were randomly selected from each of the four districts using simple random sampling. Within each selected block, fifty Anganwadi Centers were randomly selected using probability proportional to size sampling from the official AWC registry maintained by the Integrated Child Development Services department, serving as primary sampling units to ensure representativeness across village sizes.

In the final stage, households with eligible women were identified through ASHA registers and village mapping exercises. Recent mothers were recruited consecutively based on eligibility criteria until target sample sizes were reached. When multiple eligible participants were available in a single village, systematic random sampling was employed to prevent clustering effects and ensure geographic distribution within villages.

Sample size was calculated to detect a minimum odds ratio of 1.5 for key perinatal behaviors with 80% power and α = 0.05, accounting for design effects from cluster sampling (design effect = 1.4) and anticipated 15% non-response rate. This yielded a minimum required sample of 1,100 mothers and 380 ASHAs across the four districts.

#### 2.3.2 Data collection

The final sample comprised 400 ASHA surveys and 1,166 client surveys after excluding 34 for incomplete interviews. All mother respondents had given birth within six months before data collection to minimize recall bias.

All surveys were administered by trained female research assistants through face-to-face structured interviews using standardized questionnaires. Daily supervision was provided by field coordinators who reviewed completed questionnaires for completeness and conducted random quality checks through re-interviews with 5% of participants to ensure data quality.

The sample captured demographic diversity typical of the study area. Mothers ranged from 15 to 41 years (mean: 23) and ASHAs from 23 to 65 years (mean: 38). Educational levels ranged from non-literate mothers (approximately 50%) to ASHAs with a mandatory minimum of eight years of education. Religious composition reflected local demographics, with mothers being 87.4% Hindu, 11.9% Muslim, and 0.7% other, while ASHAs were 96.5% Hindu, 3% Muslim, and 0.5% other.

The survey instrument explored perinatal health behaviors and healthcare provider influences during pregnancy, labor, and postpartum periods. The study examined various perinatal behaviors using both binary and multiple-choice responses, categorizing perinatal health behaviors into biomedical behaviors and neutral behaviors. We coded each behavior with a recommended response based on established perinatal health guidelines and cultural practices (Table 1).

**Table 1:** The list of perinatal behaviors analyzed for the paper includes the recommended response and coding definition.

Behavior	Rec response	Definition
4+ Antenatal Checkups	Yes	Four or more antenatal checkups during pregnancy; yes = had 4 or more checkups, no = 0 to 3
Fast While Pregnant	No	Fasting while pregnant; yes = yes did fast in some form (regularly or for festivals), no is did not fast at all
Work While Pregnant	No	Working while pregnant; yes = frequently or sometimes, no = never
Dry Cord Care	No	Applied substance to the cord stump; yes = yes applied something (but there are many options for what was applied); no means applied nothing.
Delayed Bathing (child)	No	Bathing newborn within 24 h of birth; yes = gave bath within 24 h; no = after 24 h or bath not given
Hospital Delivery	Yes	Hospital delivery; yes = delivery in government or private hospital; no = home birth
TIBF	Yes	Timely initiation of breastfeeding; yes = breast fed within first hour; no = breast fed after first hour or never breastfed
Feed Colostrum (child)	Yes	Feed colostrum to newborn after birth; yes = fed colostrum; no = did not feed colostrum
Timely Registration (Preg)	Yes	Timely antenatal care registration; yes = registered for ante-natal care within first 3 months of pregnancy; no = registered after three months or did not register.
Avoid Cereal (postpart)	No	Avoided cereals the first week after birth; yes = avoided cereals just after delivery; no = did not avoid cereals
Iron (IFA) consumption	Yes	IFA tablets; yes = consumed the full recommended amount of iron folic acid tablets; “no” = did not consume or consumed less than recommended
Conceal Pregnancy (1st Tri)	No	Concealed the pregnancy from outsiders; yes = hid to three months or longer than three months; No means did not hide or hid for 2 months or less.
Dai Visit (pregnancy)	Yes	Dai visit in household to provide health related services/advice during pregnancy; yes = yes Dai visited; no = Dai did not visit
Abstain Sex (pregnancy)	Yes	Abstain from having physical relations with husband during pregnancy; yes = yes, avoided sexual relations; no = had sexual relations
Avoid Market (3 Tri)	Yes	Avoid going to markets/relatives/other households in the village during last trimester of the pregnancy; yes = yes avoided markets in last trimester; no = no
Consult Priest (pregnancy)	Yes	consult any priest/maulana (religious leaders) during pregnancy; yes = once, frequently, or sometimes; no = never
Dai Visit (labor)	Yes	Dai visit in household to provide health related services/advice during labor; yes = yes Dai visited; no = Dai did not visit
Dai Visit (postpartum)	Yes	Dai visit in household to provide health related services/advice during post-delivery period; yes = yes Dai visited; no = Dai did not visit
Chhathi (celebration)	Yes	Celebrate Chhathi, a social celebration on 6th day of the child’s birth; yes = yes, I celebrated Chhathi; no = I did not celebrate
Mom-NB Isolation	Yes	Child and mother kept separately from rest of the household and neighbors; yes = yes, we were kept separate; no = we were not kept separate

Biomedically Recommended Behaviors comprised twelve key behaviors directly impacting maternal and child health outcomes. These included essential healthcare practices such as completing four or more antenatal checkups, timely pregnancy registration within the first three months, and hospital delivery. The analysis also examined potentially harmful practices such as fasting or heavy work during pregnancy, applying substances to the umbilical cord stump, and bathing newborns within 24 h of birth. We documented nutritional information through variables like iron tablet consumption and colostrum feeding.

Neutral Behaviors documented eight culturally significant practices classified as having no direct positive or negative health implications based on current biomedical evidence and WHO perinatal care guidelines. This classification was developed through qualitative insights and consultation with maternal and child health specialists. These included traditional practices such as consulting Dais during pregnancy, labor, and postpartum periods, observing sexual abstinence during pregnancy, and participating in cultural celebrations like Chhathi, a naming ceremony celebrated on the sixth day after birth. Behaviors were classified as neutral when systematic reviews showed insufficient evidence of harm or benefit, distinguishing them from practices with established biomedical recommendations.

Surveys were administered using Android tablets with audio recording and built-in validation checks to flag discrepancies in real-time. Binary behavioral outcomes were first manually coded during the qualitative phase, then programmed into the survey software for systematic application. The influence categories were prepopulated based on an extensive list developed through qualitative insights and literature review, allowing respondents to spontaneously identify influencers from this comprehensive standardized list. The survey also gathered information on healthcare provider interactions and demographic characteristics.

#### 2.3.3 Instrument development and validation

We developed culturally appropriate instruments through a rigorous multi-stage process. Initial English instruments were constructed from validated tools, including the National Family Health Survey (NFHS-5) and WHO maternal health assessment guidelines, adapted for the specific research questions about healthcare provider influences.

All survey instruments and interview guides were translated into Hindi by certified translators with expertise in health terminology, as Hindi is commonly understood and spoken. Back-translation was performed by independent translators to ensure accuracy. Instruments underwent pilot testing with 30 participants in each linguistic region (90 total), with modifications made based on comprehension difficulties, cultural appropriateness concerns, and feedback on question sensitivity. Final versions were approved by community representatives and health experts. The validation process ensured instruments maintained scientific rigor while remaining culturally accessible to our target population.

### 2.4 Quality assurance and bias mitigation

We implemented several strategic measures to enhance data quality and minimize bias throughout the study. To combat recall bias, we recruited mothers within six months of delivery when pregnancy experiences remain vivid and anchored our structured interviews to memorable pregnancy milestones.

Recognizing that healthcare provider preferences and behaviors often carry cultural judgment, we addressed social desirability bias by ensuring interviews were conducted privately by experienced female field investigators from the same regional background as participants. These researchers, well-versed in local cultural nuances, established rapport while providing explicit assurances of anonymity.

To minimize interviewer bias, we implemented standardized training protocols and structured interview guides to ensure consistency across all data collectors. Research assistants participated in role-playing exercises and practice interviews during training, with feedback provided on maintaining neutrality and avoiding leading questions. Field supervisors conducted periodic observation of interviews and provided corrective feedback when needed. Additionally, the rotation of research assistants across villages helped minimize systematic interviewer effects on responses.

Recognizing that family power structures, particularly in joint family settings, might influence responses, researchers employed several mitigation strategies. When possible, FGDs were conducted with participants from similar family structures separately. For mixed groups, facilitators used indirect questioning techniques. We noted observations of family dynamics for analytical consideration.

To address potential role conflict, ASHA participants were explicitly instructed to discuss their healthcare experiences and beliefs as mothers and community members rather than in their professional capacity. Personal healthcare experiences were explored before professional practices to reduce social desirability bias, with all ASHAs interviewed individually to encourage authentic sharing of personal information.

### 2.5 Data analysis

#### 2.5.1 Mixed-methods integration

Qualitative findings informed quantitative variable construction, particularly the categorization of health behaviors and identification of key influencers to include in regression models. During analysis, quantitative patterns were interpreted using qualitative insights about mechanisms of influence. For example, statistical findings showing complementary temporal patterns between ASHAs and Dais were explained through ethnographic observations of their different roles and FGD insights about community perceptions. Discrepancies between quantitative and qualitative findings were explored through additional analysis and discussed as limitations.

#### 2.5.2 Quantitative analysis

The focus of this analysis is on the roles of ASHA and Dai, though they are among many possible influencers in health-related decision-making. Participants were asked to identify sources of influence for their health behaviors throughout the perinatal period. Influencers mentioned spontaneously by participants were recorded from a pre-populated list based on earlier focus group discussions and pilot testing. The list included ASHA, Dai, family members, government and private medical practitioners, media, and neighbors. Importantly, participants were not explicitly prompted to mention ASHA or Dai, thus mentions reflect genuine salience rather than survey priming.

We used logistic regression analyses to examine each influencer's impact on specific behaviors, considering potential interactions between influencers and behaviors. Starting from a saturated model, we applied a backward model selection procedure, dropping insignificant interactions based on AIC criteria, to arrive at the most parsimonious model (60, 61). Predicted probabilities from this model reflect the total effects, including retained interactions and control variables.

Regression models were built for each of the 19 perinatal behaviors as dependent variables. Binary coding of biomedical behaviors was based on National Health Mission guidelines and WHO recommendations. For culturally nuanced practices, detailed responses on timing and frequency were collected, then coded using standardized decision rules developed through expert consultation and validated against qualitative findings. Initial saturated models included all potential influencers, demographic controls (age, parity, education), socioeconomic status, and all two-way interactions between primary influencers and behaviors. Backward selection using AIC criteria was chosen over forward selection to avoid missing important interactions, starting from the saturated model and removing non-significant terms. Control variables were retained regardless of significance to adjust for potential confounding.

Control variables in these models included categorical measures of age, age at marriage, parity, education, and wealth. Wealth was calculated using principal component analysis on responses to a 36-item inventory about household assets and characteristics, such as toilets, housing type, and possession of items like radios and bicycles. Participants could identify multiple influencers per question.

All statistical analyses were conducted using R version 4.2.0. Descriptive statistics included frequencies, proportions, and cross-tabulations. Logistic regression models were fitted using the glm() function, with backward selection performed using the step() function based on AIC criteria. Model diagnostics included assessment of residuals, influential observations, and goodness-of-fit using Hosmer-Lemeshow tests where appropriate.

Multicollinearity was assessed using variance inflation factors calculated with the car package in R (62). We used VIF > 5.0 as the cutoff threshold, more conservative than the commonly used VIF > 10, to ensure model stability given our complex interaction terms. Variables were removed sequentially, starting with the highest VIF values. Initial assessment identified a problematic correlation between education levels and wealth quintiles (VIF > 6.0), leading to the creation of a composite socioeconomic status variable. Post-selection VIF values were all below 3.2, confirming acceptable multicollinearity levels in final models.

Model robustness was assessed through sensitivity analyses using forward selection and elastic net regularization. Results showed consistent direction and significance of main effects across methods, with backward selection chosen for final models due to superior handling of interaction terms and interpretability. Cross-validation using 10-fold CV confirmed model stability with mean prediction accuracy of 78.3% for key behaviors.

Missing data analysis showed less than 3% missing values for key variables, handled through listwise deletion given the small proportion and randomness of missingness patterns confirmed by Little's MCAR test (*p* = 0.23).

#### 2.5.3 Qualitative analysis

Qualitative and ethnographic data underwent thematic analysis to identify patterns of convergence and divergence with quantitative findings, enabling robust triangulation across methods. Analysis combined deductive and inductive approaches, with initial rapid coding using predetermined domains while remaining open to emergent themes. Two independent coders performed peer debriefing throughout the analysis, with discrepancies resolved through consensus discussion to ensure coding reliability and interpretive validity.

### 2.6 Ethical considerations

This study was approved by the Institutional Review Board at The University of Texas at Austin (Study Number: 2018-01-0027; Approval Date: February 23, 2018) and the Sigma Institutional Review Board (Study Number: 10056/IRB/D/18-19, Approval Date: December 22, 2018). Participation was voluntary, and all participants gave informed consent. The process involved explaining the study's goals, the nature of participation, and confidentiality measures. For each interview, verbal consent was documented on the consent form. This approach was chosen in line with IRB-approved protocols, considering local literacy levels and cultural norms. The consent process was conducted in Hindi, with forms translated into Hindi to facilitate understanding. Audio recordings were stored on password-protected devices and deleted after transcription verification. All transcripts were de-identified using participant codes, with physical documents secured in locked storage.

## 3 Results

Our mixed-methods analysis draws on three complementary data streams: focus group discussions with mothers and mothers-in-law and key informant interviews with community health influencers and providers [FGD][KII]; ethnographic field observations of ASHAs [ETH]; and a quantitative survey of 1,166 young mothers and 400 ASHAs^1^[QUANT]. We organized the findings around our three primary research questions, with each section integrating insights across methods to provide a comprehensive understanding of how ASHAs and Dais influence perinatal healthcare decisions in rural Bihar. Throughout the Results, we append bracketed tags—[FGD], [KII], [ETH], or [QUANT]—to each finding to indicate its data source.

### 3.1 How do ASHAs and Dais influence perinatal healthcare decisions differently?

#### 3.1.1 Distinct Spheres of Influence and Authority

Our ethnographic observations revealed fundamentally different positionings of ASHAs and Dais within the community healthcare system. ASHAs functioned primarily as formal healthcare intermediaries, operating within institutional frameworks and timelines. As one ASHA trainer explained:

“Earlier, ASHAs were not known in the village, but now every house knows them by name. Villagers recognize them as “someone's daughter-in-law” or as a ‘government worker.’ They maintain proper records and ensure families get their government benefits”. [ETH]

In contrast, Dais derived their authority from deep community roots and generational knowledge transfer, as illustrated by one Dai's account:

“We have been helping mothers deliver babies for generations. We know which herbs help with morning sickness and which positions make delivery easier. These practices have worked for our mothers and grandmothers.” [ETH]

Our analysis revealed distinct patterns in delivery location preferences and healthcare provider roles. Institutional deliveries were common, with 69.75% occurring in government hospitals, where ASHAs played a crucial intermediary role. Of these hospital deliveries, 67% were facilitated by ASHA accompaniment, primarily for navigating hospital procedures and managing documentation for incentives and certificates. Though home births constituted just 17.4% of all deliveries, Dais maintained a dominant presence in this space, conducting nearly 90% of these births. [QUANT].

This bifurcation in delivery settings revealed an interesting contrast in how communities perceived provider expertise: while ASHAs are valued for their institutional knowledge and abilities to navigate the formal healthcare system, Dais commanded respect for their experiential knowledge of childbirth practices.

#### 3.1.2 Temporal Patterns of Engagement

**Figure 1:**
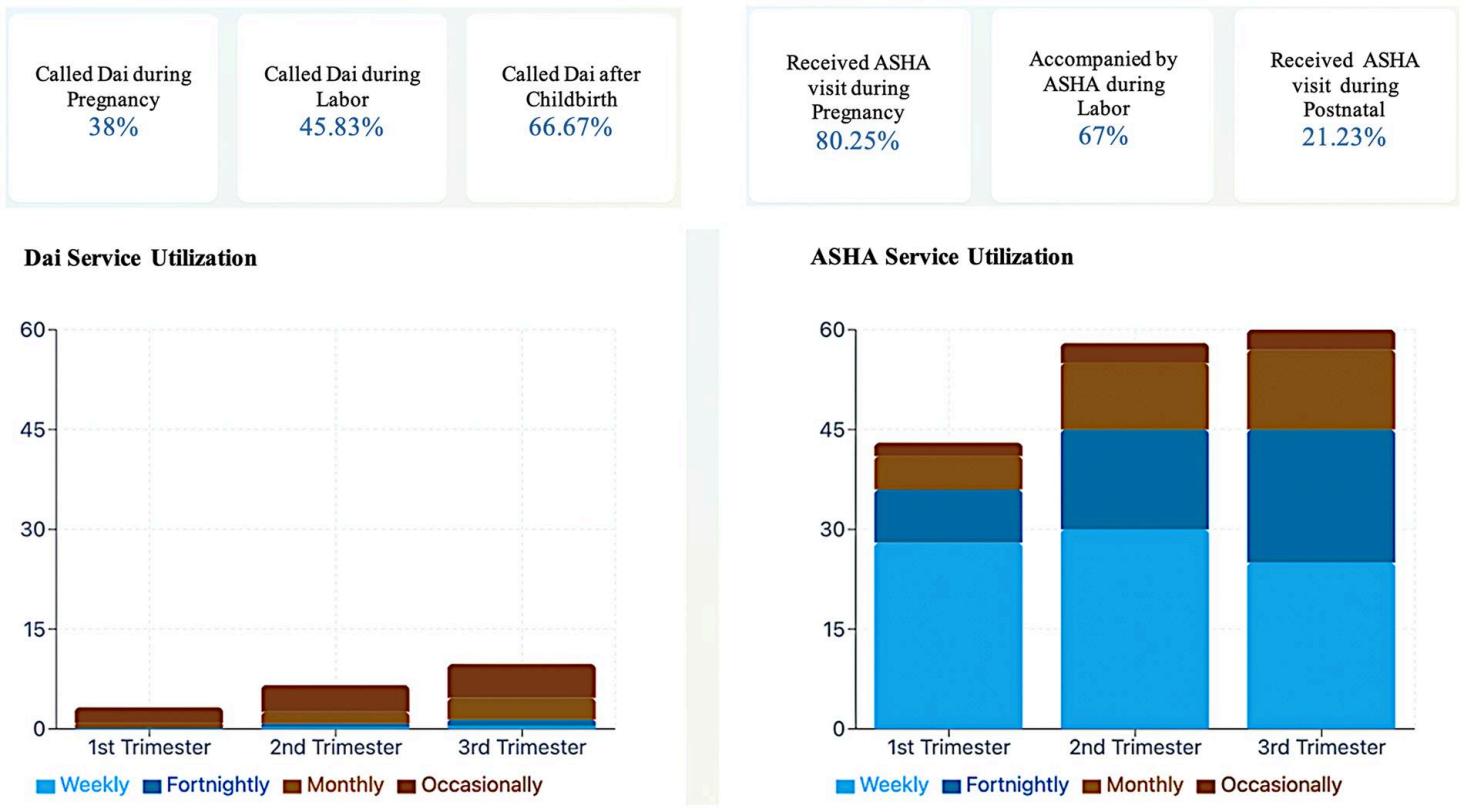
Frequency of home visits and primary services provided by ASHAs and Dais during the perinatal period.

Our quantitative analysis revealed distinct and complementary temporal patterns in women engaging with ASHA workers and Dais across the perinatal period. Among the 1,166 surveyed mothers, ASHA involvement was highest during pregnancy (80.25%) and labor (67%) but decreased significantly in the postpartum period (21.33%). In contrast, Dai involvement showed a progressive increase from pregnancy through postpartum: 34.83% received Dai visits during pregnancy, rising to 45.83% during labor and reaching 66.67% in the postpartum period (Figure 1). [QUANT].

The frequency of visits showed distinct patterns across trimesters, particularly highlighting differences between ASHAs and Dais. ASHA workers maintained consistently high weekly visit rates throughout pregnancy, peaking in the second trimester. In contrast, interactions with Dai were notably limited during pregnancy, with low visit rates in the first trimester (3.24%), a slight increase in the second trimester (6.58%), and reaching just 9.75% in the third trimester. Dai involvement significantly intensified only during labor and postnatal care periods.

Additional analysis of ASHA survey data (*N* = 400) provides further insight into these temporal engagement patterns. In prenatal contexts, 29.5% of ASHAs reported spending time with Dais, rising to 46.75% during delivery and 52.25% in postnatal visits. Notably, 19% indicated they did not spend time with Dais, while 5.25% of ASHAs stated that no Dai was present in their village. This progressive increase in ASHA-Dai interaction mirrors the earlier observed pattern of Dai involvement with mothers, suggesting a systematic shift in care dynamics across the perinatal period. ASHAs reported an average of 2.15 interactions with Dais per month, ranging from 0 to 15 encounters, indicating substantial variability in collaborative practices across communities. [QUANT].

Young mothers in group discussions illuminated the rationale behind this complementary utilization:

“ Before going to the hospital for delivery, DAI is called to confirm her labor pain” [FGD]

These findings highlight the complementary nature of the roles of ASHAs and Dais rather than a competitive dynamic. While ASHAs primarily handle formal healthcare tasks during pregnancy and labor, Dais become increasingly active in areas that formal services cover less thoroughly, particularly during the postpartum period.

#### 3.1.3 Differential Patterns of Influence

Our ethnographic observations initially revealed a crucial distinction in how ASHAs and Dais influenced perinatal health behaviors, with participants characterizing ASHAs as “outside the home” actors and Dais as “inside the home” care providers. This qualitative insight led us to conduct a detailed statistical analysis of behavioral influence patterns, which provided strong quantitative support for this inside-outside dichotomy while revealing additional nuances in how these providers shape perinatal healthcare decisions.

**Figure 2:**
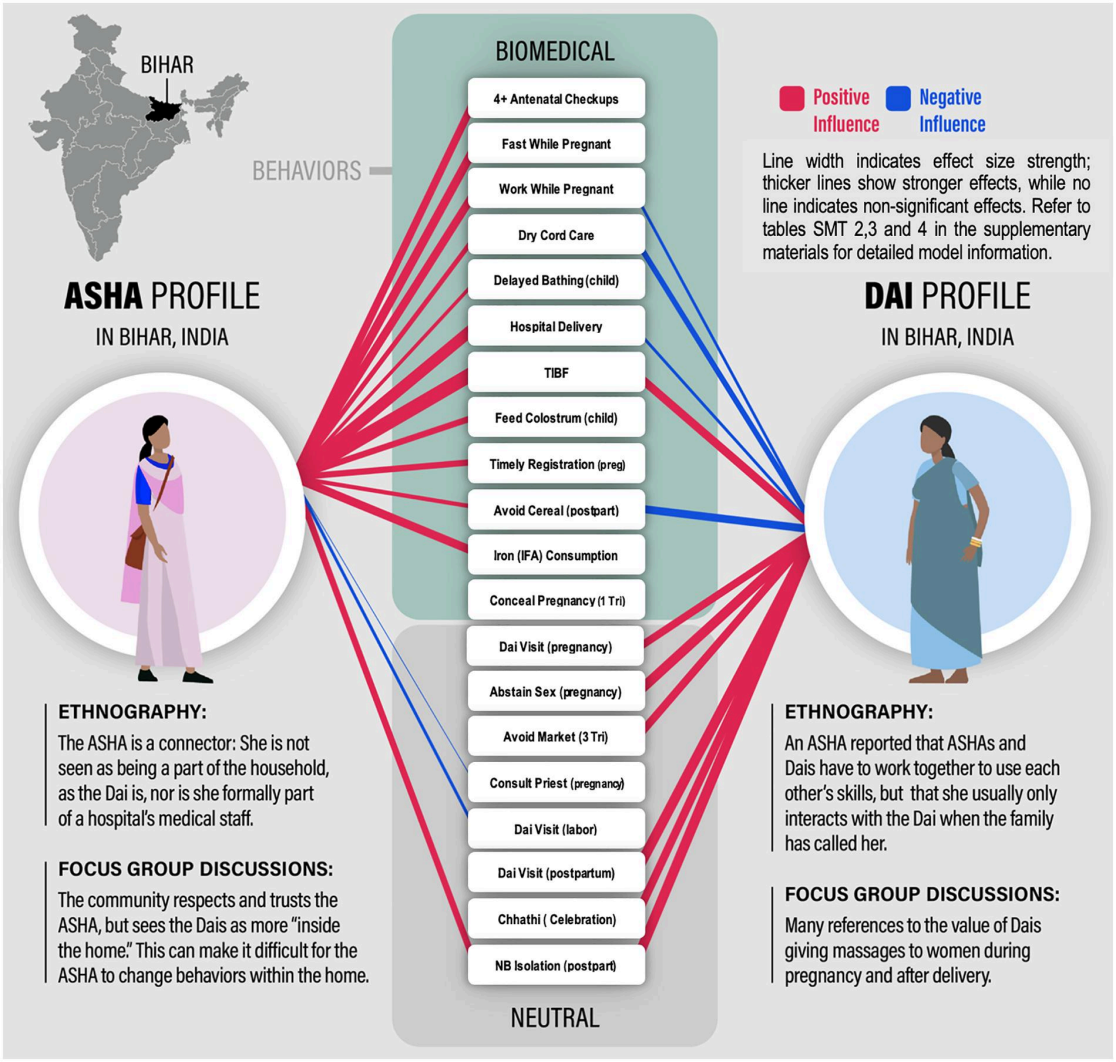
Patterns of ASHA and Dai influence on perinatal behaviors in Bihar, India. Red lines indicate positive effects; blue lines indicate negative effects. Line thickness corresponds to statistically significant predicted probabilities.

Table 2 summarizes the influence of ASHAs and Dais on 19 perinatal behaviors using selected logistic regression models, which include effect sizes and standard errors while accounting for controls, influencers, behaviors, and influencer × behavior interactions. Additionally, Figure 2 visualizes the modeled effects of ASHAs and Dais across these behaviors, with line thickness and color indicating the likelihood of adoption. The analysis indicates that ASHAs most strongly influenced behaviors associated with formal healthcare institutions. Detailed statistical models and their results are available in the Supplementary Materials^2^. For additional information about the analytical procedure, please refer to the Quantitative Methods subsection within the Methods section. For instance, women who identified ASHAs as influencers showed markedly higher probabilities of completing four or more antenatal checkups (*β* = 0.537, *SE* = 0.040) and opting for hospital delivery (*β* = 0.905, *SE* = 0.014). This pattern aligns with our ethnographic observations of ASHAs functioning as institutional intermediaries, helping women navigate formal healthcare systems (Table 2). [QUANT].

**Table 2:** Summary results of selected logistic regression model that includes controls, influencers, behaviors, and influencer × behavior interactions.

Influencer	Question	Fit coefficient (β*)*	Standard error (SE)	N	Effect	Type
ASHA	Conceal Pregnancy (1 Trimester)	0.234	0.041	183	Negligible	biomed
Dai	Conceal Pregnancy (1 Trimester)	0.080	0.016	3	Negligible	biomed
ASHA	Timely Registration (preg)	0.736	0.028	521	Positive	biomed
Dai	Timely Registration (preg)	0.080	0.016	1	Negligible	biomed
ASHA	4+ Antenatal Checkups	0.537	0.040	357	Positive	biomed
Dai	4+ Antenatal Checkups	0.110	0.020	0	Negligible	biomed
ASHA	Fast While Pregnant	0.880	0.054	40	Positive	biomed
Dai	Fast While Pregnant	0.350	0.046	1	Negligible	biomed
ASHA	Work While Pregnant	0.722	0.060	176	Positive	biomed
Dai	Work While Pregnant	0.238	0.037	6	Negligible	biomed
ASHA	Iron (IFA) Consumption	0.433	0.044	331	Positive	biomed
Dai	Iron (IFA) Consumption	0.030	0.006	1	Negligible	biomed
ASHA	Hospital Delivery	0.905	0.014	685	Positive	biomed
Dai	Hospital Delivery	0.235	0.037	38	Negligible	biomed
ASHA	Feed Colostrum (child)	0.938	0.016	481	Positive	biomed
Dai	Feed Colostrum (child)	0.706	0.063	91	Negligible	biomed
ASHA	Timely Initiation of Breastfeeding (TIBF)	0.827	0.037	448	Positive	biomed
Dai	Timely Initiation of Breastfeeding (TIBF)	0.573	0.083	85	Positive	biomed
ASHA	Dry Cord Care	0.699	0.039	332	Positive	biomed
Dai	Dry Cord Care	0.203	0.049	113	Negative	biomed
ASHA	Delayed Bathing (child)	0.880	0.027	296	Positive	biomed
Dai	Delayed Bathing (child)	0.556	0.048	199	Negligible	biomed
ASHA	Avoid Cereal (postpartum)	0.922	0.023	178	Positive	biomed
Dai	Avoid Cereal (postpartum)	0.398	0.063	100	Negative	biomed
ASHA	Consult Priest	0.013	0.010	31	Negative	neutral
ASHA	Abstain Sex (pregnancy)	0.252	0.037	116	Negligible	neutral
Dai	Abstain Sex (pregnancy)	0.672	0.085	5	Positive	neutral
ASHA	Avoid Market (3rd Trimester)	0.587	0.047	91	Negligible	neutral
Dai	Avoid Market (3rd Trimester)	0.896	0.035	6	Positive	neutral
ASHA	Dai Visit (pregnancy)	0.123	0.023	56	Negligible	neutral
Dai	Dai Visit (pregnancy)	0.460	0.097	6	Positive	neutral
ASHA	Dai Visit (labor)	0.078	0.018	163	Negative	neutral
ASHA	Dai Visit (postpartum)	0.474	0.087	115	Negligible	neutral
Dai	Dai Visit (postpartum)	0.762	0.071	34	Positive	neutral
ASHA	Mom-Newborn (NB)_Isolation	0.572	0.097	119	Positive	neutral
Dai	Mom-Newborn (NB)_Isolation	0.745	0.075	58	Positive	neutral
ASHA	Chhathi (Birth Celebration on 6th day postpartum)	0.568	0.053	29	Negligible	neutral
Dai	Chhathi (Birth Celebration on 6th day postpartum)	0.889	0.039	15	Positive	neutral

The quantitative data also illuminated the depth of Dais' influence within domestic spaces. Their strongest effects appeared in traditional postpartum practices, with notably high predicted probabilities for “Chhathi”, a birth celebration on the sixth day postpartum (*β* = 0.889, *SE* = 0.053), and newborn isolation practices (*β* = 0.745, *SE* = 0.075) (Table 2). [QUANT]

These findings support our key informant discussions, during which Dais described how their role complements the biomedical healthcare system:

“After the doctor leaves, my work begins. ASHA does the official paper; I do the massaging and check the new mother's condition”. [KII]

Particularly interesting were the areas where the influence of ASHAs and Dais created opposing effects. In cord care practices, for example, ASHA influence significantly decreased the likelihood of traditional applications (*β* = 0.699; *SE* = 0.039), while Dai influence promoted these practices (*β* = 0.203; *SE* = 0.049) (Table 2). This quantitative evidence helps explain the negotiation patterns we observed in our ethnographic work, where families often had to navigate contradictory recommendations from different providers.

The analysis also revealed rare instances of synergistic influence, particularly in the timely initiation of breastfeeding (ASHA_*β* = 0.827, *SE* = 0.037; Dai_*β* = 0.573; *SE* = 0.083) and mother-newborn isolation (ASHA_*β* = 0.572, *SE* = 0.097; Dai_*β* = 0.745; *SE* = 0.075) (Table 2). [QUANT].

These findings support our qualitative observations of emerging collaborative patterns between traditional and biomedical healthcare systems. As one Dai noted:

“When we see signs of danger like excessive bleeding or breech position, we now tell families to go to the hospital. Times have changed, and some complications need doctor's care”. [ETH]

This integration of quantitative and qualitative evidence reveals how the inside/outside distinction manifests in measurable behavioral outcomes while illuminating the mechanisms through which these influences operate. The statistical patterns strengthen our understanding of how traditional norms and institutional boundaries shape provider influence, while our ethnographic data helps explain why these patterns emerge and how communities navigate them.

### 3.2 How do ASHA and Dai exercise their influence?

#### 3.2.1 Influence Mechanisms of ASHA Versus Dais

Our research revealed distinct yet complementary mechanisms through which ASHAs and Dais influence perinatal healthcare, reflecting broader patterns in how communities engage with biomedical and traditional healthcare systems. ASHAs exercised influence primarily through public health channels and structured health education approaches. In contrast, Dais operated through traditional authority and direct physical care, creating a complex interplay between institutional authority and traditional knowledge.

Ethnographic observations documented ASHAs’ crucial role in navigating healthcare bureaucracy, as illustrated by one field note:

“SMA01 stands next to her patients in the lab, adjusting their hand positions for the blood test. She also asks family members to reserve beds in the post-op room quickly. Otherwise, someone else might take the space”. [ETH]

The quantitative data strongly supported this institutional role, with ASHAs significantly influencing formal healthcare utilization. Women who reported ASHA influence were more likely to opt for hospital delivery (*β* = 0.905; *SE* = 0.014) and recommended four or more antenatal checkups (*β* = 0.537; *SE* = 0.040). Survey data further revealed ASHAs' interactions with traditional care systems, which primarily focused on specific health outcomes, with the highest engagement in newborn care (17.8%), maternal care (17.8%), and delivery practices (14.6%) while maintaining limited involvement in broader social or family matters (3.8% and 10.1% respectively). [QUANT].

In contrast, Dais wielded influence through hands-on engagement and cultural expertise, as evidenced by our analysis of postpartum services (Table 3). Among mothers receiving Dai care, 75.67% received maternal massage services, and 67.42% had their newborns massaged by the Dai. The comprehensive nature of Dai care extended beyond physical services to include dietary guidance (58.33%), ritual bath services (27.92%), and traditional healing practices (16.92%). [QUANT].

**Table 3:** Percentage of women (*N* = 800) receiving traditional postnatal care services provided by Dais during the postnatal period.

Service	Percentage of women receiving service (%)
Maternal Massage (Post-delivery body massage)	75.67
Newborn Massage (Daily infant massage)	67.42
Ritual Bathing for Newborns (Ceremonial baths)	27.92
Dietary Guidance (Traditional dietary advice)	58.33
Traditional Healing Practices (Remedies, herbs)	16.92

“N” indicates the total number of women who received visits from traditional birth attendants (Dais) during the postnatal (neonatal) period. The services listed represent commonly provided traditional care practices.

Focus groups with mothers revealed the deep trust communities placed in Dais' experiential knowledge:

“The Dai says if the child is bathed too early, it might “anger the ancestors”. If families skip certain rituals, they risk the child's health. She insists that's why she comes every day for six days to massage the mother and newborn”. [FGD]

These contrasting influence mechanisms reflect a broader pattern in how communities engage with healthcare providers. ASHAs' influence operates primarily through formal channels, leveraging institutional authority and systematic health education approaches. Their effectiveness is particularly evident in increasing institutional delivery rates and preventive care adherence. Dais, meanwhile, exercise influence through deeply embedded traditional health practices and direct physical care, maintaining authority through generational knowledge and hands-on engagement with mothers and newborns.

#### 3.2.2 Communication and Trust-Building Strategies

Each provider group employed distinct approaches to building trust and communicating health messages. ASHAs relied heavily on formal tools and structured interactions:

“We use flipcharts and mobile apps to explain the importance of iron tablets and proper nutrition. We organize group meetings where women can learn and ask questions”. [KII]

In contrast, Dais utilized personal relationships and cultural understanding:

“I examine the pregnant woman, and the ASHA does her documentation. I’m not involved in the government forms or incentives. My work is to see the mother, do massages, and check if everything is okay”. [KII]

Recent survey data underscores the variety of topics ASHAs and Dais discuss when coordinating services. Among the ASHAs interacting with Dais, 17.8% reported conversations about newborn care, 17.8% about maternal care, and 14.6% about delivery details. Meanwhile, a smaller subset of ASHAs (10.1%) mentioned discussing broader home and family issues with Dais, highlighting that the lines between purely medical advice and socio-familial support can blur. [QUANT].

These findings shed light on the nature of collaboration and communication in the outreach. Even though ASHAs represent institutional structures, they increasingly engage in culturally resonant conversations—often with Dais as cultural gatekeepers—to ensure that recommended healthcare measures align with household norms.

### 3.3 How do communities navigate between competing recommendations?

#### 3.3.1 Integration Patterns

Our analysis revealed sophisticated decision-making patterns through which families navigate and reconcile competing healthcare recommendations, with integration strategies shaped by practical considerations and family power dynamics. Ethnographic observations documented how women strategically blend traditional and biomedical practices based on family preferences and perceived efficacy:

“One mother says, ‘I’ll take the iron tablets but will also keep the fire pot near the child—just in case. I don’t want to anger my elders”. [ETH]

Quantitative data reflected this selective adoption approach, where high rates of institutional delivery (82.6%) and traditional postpartum practices (78.4%) demonstrated families' active engagement in creating hybrid care models that satisfied medical and cultural requirements. [QUANT].

However, the implementation of these hybrid approaches was heavily mediated by family power structures, particularly in joint family settings. Mothers' focus groups revealed how women often navigate healthcare decisions within complex family dynamics:

“One woman says she can’t get any rest at her marital home. She comes to her mother's house for the sterilization surgery because her marital side doesn’t support her, and there's no one to look after her”. [FGD]

The quantitative data reinforced this finding, showing that women's healthcare choices were significantly influenced by family gatekeepers, with mother-in-law's preferences frequently determining the balance between traditional and biomedical practices. These intertwined patterns of care integration and family decision-making reveal a nuanced process through which communities reconcile competing healthcare recommendations. Rather than simply choosing between traditional and biomedical approaches, families engage in careful negotiation that considers both medical efficacy and family harmony.

#### 3.3.2 2 Areas of Complementarity and Conflict

Our analysis identified specific areas where ASHA and Dai's influences reinforced each other, particularly in the timely initiation of breastfeeding (65.3% compliance) and mother-newborn isolation (80.6% adherence). [QUANT]

However, tensions emerged around certain practices, as illustrated by this focus group observation with mothers:

“They [the well-off family] told the ASHA, ‘We get info from YouTube; we don’t need your suggestions on breastfeeding.’ The ASHA felt hurt but said she’ll still help when it's time for immunizations”. [FGD]

Competing recommendations were particularly evident in postpartum practices. While ASHAs promoted immediate postpartum nutrition through regular food consumption, Dais often advocated for traditional dietary restrictions:

“The mother says she is on a diet of “milk and ginger halwa” only and will not eat grains before the sixth day. The ASHA tells her to eat rice and pulses, or else she’ll weaken, but the mother refuses until Chhatti”. [ETH]

The ASHA survey data revealed specific areas of tension, with 21% reporting direct work-related conflicts with Dais. The nature of these conflicts often centered on reputational concerns (65.8% of reported conflicts) and the creation of obstacles to formal healthcare delivery (47.7%). However, the data also revealed constructive conflict resolution approaches, with 73.4% of ASHAs addressing conflicts through direct discussion with Dais, suggesting an emerging framework for negotiating between traditional and biomedical healthcare tensions. [QUANT].

The survey also provided insight into how ASHAs view Dai services, with 50% acknowledging Dais' role in providing pregnancy massages, 49% in labor confirmation, and 49.4% in cord cutting. This recognition of Dai services by ASHAs suggests a nuanced understanding of traditional care practices, even as they work to promote institutional healthcare. Notably, 23.1% of ASHAs reported that Dais actively assisted with institutional deliveries, indicating emerging areas of practical cooperation despite broader systemic tensions. [QUANT]

#### 3.3.3 Adaptation and Evolution

Both provider groups showed evidence of adaptation to changing healthcare landscapes. Dais increasingly acknowledged the importance of institutional delivery for complicated cases:

“When we see signs of danger like excessive bleeding or breech position, we now tell families to go to the hospital. Times have changed, and some complications need doctor's care”. [KII]

Similarly, ASHAs demonstrated flexibility in accommodating traditional practices when they didn't conflict with essential medical care:

“Some traditional herbs for nausea are fine as long as they don’t replace the prescribed medications. We need to respect these practices while ensuring mother's safety”. [ETH]

These patterns suggest that communities have developed sophisticated strategies for integrating traditional and modern healthcare recommendations, creating a hybrid model of perinatal care that draws on both systems' strengths while navigating their contradictions.

## 4 Discussion

The intersection of traditional and biomedical healthcare systems represents a critical frontier in global perinatal healthcare delivery. This study examined this intersection through a comparative analysis of ASHAs and Dais in rural Bihar, India, revealing complex patterns of interaction that challenge the conventional understanding of how parallel healthcare systems coexist and influence perinatal health behaviors. Our findings extend beyond earlier observations of healthcare pluralism and demonstrate that the progress in rural healthcare is not a simple trajectory of traditional practices giving way to biomedicine but rather presents a deeply layered landscape where traditional wisdom and biomedical knowledge coexist in ways that defy linear progression (17).

### 4.1 Distinct spheres of influence in perinatal healthcare

Our first research question examined variation in how ASHAs and Dais influence perinatal healthcare decisions. The findings reveal a healthcare landscape characterized by complementary rather than competitive provider relationships, though this complementarity transcends simple “inside/outside” or “traditional/biomedical” dichotomies. Our data also reveal fluidity in these roles—Dais assist in institutional deliveries while ASHAs incorporate traditional concepts in their counseling. This observation extends beyond previous research, showing how traditional health practices and modern medical protocols often operate in parallel, demonstrating how these systems actively shape and influence each other (10).

The spatial and temporal distribution of provider influence suggests carefully negotiated rather than fixed authority boundaries. ASHAs have established primacy in formal healthcare spaces, particularly facilitating access to institutional services and promoting biomedical recommendations. Yet they function as facilitators and mediators, switching between biomedical and traditional health paradigms as needed. Their influence is strongest in areas requiring the navigation of formal healthcare systems, reflecting their role as intermediaries between communities and institutions (25). This finding supports earlier observations about ASHAs' effectiveness in improving perinatal health indicators and increasing institutional deliveries (34).

Notably, Dais maintain significant influence within domestic spaces, particularly in areas touching on traditional health practices and services. Yet this influence is not confined to the home, and sometimes Dais also extend their support into clinical settings when families request their presence during childbirth. Their authority extends beyond simple healthcare delivery to encompass broader social and cultural roles, aligning with previous research highlighting their role as cultural authorities and keepers of traditional knowledge (28). However, our findings challenge the perception that Dais are becoming obsolete due to healthcare transformation, instead demonstrating their continued relevance as evolving practitioners who increasingly navigate both traditional and biomedical systems (30). Recognizing the enduring relevance of Dais, policymakers have an opportunity to harness this strength by fostering collaborative relationships between ASHAs and Dais. Structured efforts to integrate Dais within health outreach programs could facilitate improved health messaging and strengthen overall community trust and participation.

### 4.2 Mechanisms of healthcare influence and authority

Our study of how ASHAs and Dais exercise their influence reveals distinct but complementary approaches to building and maintaining authority. These findings extend beyond previous research on ASHA effectiveness by demonstrating how their success often depends on their ability to navigate modern healthcare protocols and deeply ingrained traditional health practices (37). The effectiveness of ASHAs in promoting institutional healthcare utilization validates earlier findings about the program's success (35). However, our research reveals that their influence extends beyond simple service facilitation, challenging previous assumptions about the limited scope of their impact. This broader influence demonstrates how community health workers can adapt their roles while maintaining their institutional mandate (23). The persistence of Dai influence, particularly in postpartum care, supports previous research about the enduring value of traditional healthcare systems (21). However, our findings demonstrate how Dais actively adapt their practices to changing healthcare landscapes while maintaining their cultural authority.

Furthermore, rather than perceiving the relationship between ASHAs and Dais as inherently negative or problematic, as some literature has indicated, this study underscores the potential for this relationship to serve as a powerful vehicle for health messaging and integration (54). While policymakers may find it challenging to directly influence the practices of traditional birth attendants due to their informal and culturally embedded roles, they could nevertheless advocate for strategic alignment and cooperation between ASHAs and Dais. Collaborative training workshops, joint community outreach activities, and structured communication channels could foster mutual respect, shared learning, and a more coherent perinatal health messaging strategy.

### 4.3 Community navigation of healthcare systems

Our findings about how communities navigate between competing recommendations reveal more sophisticated patterns than previously documented. While earlier research has noted that healthcare decisions are deeply embedded in cultural and religious beliefs, our findings demonstrate how communities actively synthesize different forms of healthcare knowledge to create integrated practices (14). Women demonstrate considerable agency in this process, strategically leveraging different family relationships and healthcare options to meet their needs while maintaining social cohesion. This navigation occurs within India's caste and gender hierarchies: Dais—often from hereditary midwifery lineages—may encounter caste-based acceptance issues, while ASHAs, despite being locally appointed, can differ in caste from Dais or clients (34, 55). These roles influence trust and authority; for instance, higher-caste families may dismiss a lower-caste Dai but accept an ASHA's advice because of her official position. Intra-household power, especially that of mothers-in-law, further mediates whose advice is heeded. Although we did not stratify results by caste or class, we note these dynamics to contextualize interpretation. Accordingly, efficacious perinatal interventions should weigh medical validity alongside fit with family decision frameworks and valued traditions and can productively promote hybrid approaches that honor cultural meaning while emphasizing beneficial biomedical practices, thereby reducing perceived conflicts between systems (52).

### 4.4 Limitations and future research directions

Several limitations of our study are relevant to interpreting these findings. First, while our mixed-methods approach provided rich insights into provider influence patterns, the cross-sectional nature of the data limits our ability to track how these patterns evolve. Longitudinal studies would be valuable for understanding the dynamic nature of these relationships. Second, while providing deep contextual understanding, our focus on Bihar may limit the generalizability of specific findings to other cultural and institutional contexts. Future research in different settings would help identify which aspects of our findings reflect broader patterns of healthcare integration and which are specific to particular cultural contexts. Third, the complexity of social systems makes it challenging to isolate specific causal pathways in provider influence mechanisms. More targeted interventional studies could help clarify the relative impact of different influence mechanisms and identify specific factors that facilitate or hinder successful healthcare integration. Fourth, Social desirability bias may have influenced self-reported behaviors, particularly around adherence to biomedical recommendations or traditional practices viewed negatively by different community segments. While we employed indirect questioning and private interview settings, some participants may have provided responses they perceived as socially acceptable rather than accurate. Selection bias could have occurred if certain population subgroups were systematically less likely to participate, though our high response rate (89.2%) suggests minimal impact*.* We acknowledge binary coding may obscure the partial and mixed practices common in rural Bihar (e.g., feeding colostrum yet giving prelacteal; selectively following dietary restrictions); future work should use other measures to capture these nuances of adoption. Additionally, practical trials that implement and evaluate collaborative interventions between ASHAs and Dais could yield valuable insights into effective strategies for healthcare integration.

## 5 Conclusion

This research reveals how perinatal healthcare in rural communities operates through sophisticated systems of complementarity rather than simple competition between traditional and biomedical providers (20). The findings challenge the narrative of inevitable traditional practice displacement and oversimplified models of biomedical pluralism. Instead, they suggest a dynamic process of healthcare evolution where different systems of knowledge and practice actively shape each other while maintaining distinct spheres of influence.

The observed patterns of provider adaptation and community navigation between different healthcare paradigms suggest promising pathways for developing more integrated approaches to perinatal healthcare. Rather than viewing traditional and biomedical healthcare systems as inherently antagonistic, policymakers and healthcare planners might focus more productively on understanding and supporting the organic processes of integration already occurring at the community level. The success of communities in integrating different forms of healthcare suggests the potential for developing more culturally responsive approaches to perinatal healthcare delivery. Such approaches would recognize and build upon existing patterns of healthcare pluralism while addressing areas where better integration could improve health outcomes. The distinct temporal patterns of provider engagement suggest opportunities for more targeted healthcare interventions, particularly leveraging Dais' involvement in postpartum care to address gaps in institutional healthcare coverage (56).

The observed patterns of provider adaptation indicate potential pathways for healthcare system integration that preserve valuable aspects of both traditional and biomedical care while addressing areas of conflict (57). The willingness of both ASHAs and Dais to modify their practices in response to changing healthcare needs suggests the potential for more formal integration strategies that build on these organic adaptations (42). Beyond perinatal healthcare, these findings have broader implications for domains where traditional and biomedical influencers coexist, such as education, environmental sustainability, and financial inclusion. In these contexts, similar patterns of adaptation and negotiation between knowledge systems can inform more effective integration strategies (58). Recognizing and valuing traditional wisdom alongside biomedical advancements can lead to more sustainable and culturally embedded solutions, fostering collaboration rather than conflict between paradigms.

A synergistic approach to integrating traditional and biomedical systems involves recognizing the dynamic interplay between historical knowledge and contemporary advancements. Successfully navigating multiple healthcare systems requires designing frameworks that promote flexibility, inclusivity, and responsiveness to local contexts. Encouraging interdisciplinary collaborations, fostering community engagement, and implementing adaptive policies can cultivate environments where traditional and biomedical influences coexist and enhance each other (45, 59). When applied systematically, such approaches can lead to more holistic and resilient solutions across various fields.

## Data Availability

The datasets presented in this study can be found in online repositories. The names of the repository/repositories and accession number(s) can be found below: https://osf.io/83n9h.

## Footnotes

^1^In the results section, we used the abbreviations [FGD] for Focus Group Discussions, [KII] for Key Informant Interviews, and [ETH] for Focused Ethnography to indicate the source of insights. All descriptive and advanced statistical results presented are derived from the quantitative survey. Detailed findings from qualitative data (FGDs and KIIs) are provided in the Supplementary Materials in Appendix II (pages 6 to 13). Comprehensive details on the Focused Ethnography findings are available in Supplementary Appendix III (pages 14 to 20). Additional details from the quantitative survey findings can be found in Supplementary Appendix IV (pages 21 to 27).

^2^The supplementary materials (Supplementary Appendix IV) provide further details on the statistical models and their specific values. Specifically, see SMT 2 for the Logistic Regression Analysis on ASHA-Dai Influence (page 22), SMT 3 for the Regression Model including All Influences, Controls, and Behavior Interactions (page 23), and SMT 4 for the Moderation Analysis including Controls and Interaction between Moderators (page 26).
