# Supplementary material for: Coexisting traditional and biomedical healthcare systems: a mixed-methods analysis of community health workers and traditional birth attendants' contributions to perinatal health behaviors in rural India
Source: Front Health Serv. 2025 Dec 18;5:1623426. doi: 10.3389/frhs.2025.1623426 (PMC12756444; doi:10.3389/frhs.2025.1623426)
Supplement: Supplementary file 1 [file Datasheet1.docx]

**Frontier in Public Health**

**SUPPLEMENTAL**

**MATERIALS**

to article

# **Coexisting Traditional and Biomedical Healthcare Systems: A Mixed-Methods Analysis of Community Health Workers and Traditional Birth Attendants' Contributions to Perinatal Health Behaviors in Rural India**

**Table of Contents - Supplementary Material**

Page No.

3

3

4

4

6

6

6

7

8

9

9

10

10

11

14

14

14

14

15

17

18

19

20

21

21

22

23

26

**Appendix I. Overview**

A. Background and Context

B. Research Questions

C. Methodology Summary

**Appendix II. Qualitative Findings (FGDs and KIIs)**

A. Focus Group Discussions Findings

1. Influence on Perinatal Healthcare Decisions

1a. Temporal Domains of Influence

1b. Spheres of Authority

2. Mechanisms of Influence

2a. Dais' Mechanisms of Influence

2b. ASHAs' Mechanisms of Influence

3. Community Navigation Between Competing Recommendations

3a. Integration Patterns

3b. Decision-Making Factors

B. Key In-Depth Interviews Findings

1. Divergent Approaches to Perinatal Care

2. Mechanisms of Influence

3. Community Navigation Between Competing Recommendations

C. Comparative Analysis of ASHAs and Dais in Rural Bihar

**Appendix III. Focus Ethnography Findings**

A. Familiarization and Overall Context

B. Major Themes and Sub-Themes

1. Contrasting Roles and Identities of ASHAs vs. Dais

1a. Position in the Community

1b. Training and Knowledge Sources

2. Mechanisms of Influence

2a. Persuasion, Trust, and Emotional Appeals

2b. Formal vs. Informal Negotiation

3. Tensions and Synergies Between ASHAs and Dais

3a. Competing Recommendations

3b. Collaborative Practices or Overlaps

4. Community Navigation and Decision-Making

4a. Familial Gatekeepers

4b. Women's Agency and Selective Adoption

5. Outcomes and Shifts in Perinatal Behaviors

4a. Adoption of Biomedical Care

5b. Persistence of Traditional Practices

C. Addressing Research Questions Directly

**Appendix IV. Quantitative Findings**

SMT 1. Descriptive Statistics of Health Behaviors

SMT 2. Logistic Regression Analysis: ASHA-Dai Influence

SMT 3. Regression Model of All Influencers, Controls, and Behavior Interactions

SMT 4. Moderation Analysis with Controls and Interaction between Moderators

**Appendix I. Overview**

**A. Background and Context**

**1. Project RISE Initiative**

This study is part of Project RISE (Ritual Innovation for Supporting Engagement), a mixed-methods collaborative project aimed at harnessing the power of ritual to improve the efficacy of Accredited Social Health Activists (ASHAs) in Bihar, India. Project RISE represents a novel approach for identifying strategies to improve ASHA motivation and performance in the context of perinatal health care delivery. While ASHAs engage in various health-related initiatives, the primary health focus of this project is their contribution to maternal and newborn health outcomes.

**2. ASHA Program in Bihar**

ASHAs constitute one of the most numerous cadres of community health workers globally. Bihar alone has approximately 100,000 ASHAs, while India has nearly one million nationwide. These frontline health workers serve as crucial links between rural communities and the formal healthcare system, particularly in regions with limited healthcare infrastructure.

**3. Project’s Goals**

The primary goals of Project RISE include:

- Understanding the complex cultural ecology surrounding ASHAs, women of reproductive age, and other health influencers
- Utilizing this knowledge to devise strategies for sustainable improvements in ASHA service quality by enhancing motivation and ability
- Documenting behaviors and rituals surrounding pregnancy and childbirth to identify target behaviors with the greatest potential for health improvement
- Mapping key touchpoints between ASHAs and beneficiaries within the maternal health space
- Developing generalizable methodological and conceptual tools applicable to other community health worker programs globally

Project RISE approaches this complex system using a nested model of interactions to document the cultural ecology of health surrounding perinatal decisions and experiences in Bihar, with particular attention to capturing embeddedness, lived experience, and connections among sources of influence and beliefs.

**B. Research Questions for the Study**

**1. Comparative Influence of ASHAs and Dais**

How do ASHAs and Dais (traditional birth attendants) differ in their influence on perinatal healthcare decisions within rural Indian communities?

**2. Mechanisms of Authority**

Through what mechanisms do ASHAs and Dais exert their authority or guidance in maternal and newborn health contexts?

**3. Community Reconciliation of Recommendations**

How do communities navigate and reconcile potentially conflicting recommendations from these two influential groups?

By addressing these questions, we aim to offer actionable insights into how leveraging both traditional and biomedical healthcare systems can improve maternal and infant well-being in a culturally resonant and effective manner.

**C. Methodology Summary**

**1. Focus Group Discussions (FGDs)**

We conducted 40 semi-structured group interviews with participants sharing similar backgrounds or experiences: 20 with recent mothers and 20 with mothers-in-law. These discussions were designed to capture ideas, opinions, and beliefs about perinatal health practices.

**2. Key Informant Interviews (KIIs)**

We completed 50 in-depth interviews with key health influencers who could not be readily recruited into group formats, including 12 ASHAs, 11 Anganwadi Workers (AWWs), 10 Dais, 6 Rural Medical Practitioners (RMPs), 5 Pandits, and 6 Mulanas.

**3. Ethnographic Fieldwork**

Our ethnographic research emphasized depth over breadth, employing intensive engagement with key informants to capture cultural nuances and shared practices. This component was conducted by a trained cultural anthropologist and two research assistants who worked in Samastipur district, Bihar, for approximately six weeks, seeking novel insights and nuanced perspectives on perinatal practices.

**4. Quantitative Survey**

We administered comprehensive questionnaires covering demographic factors, health behaviors, and perceptions of health systems and providers. Surveys were conducted with 400 ASHAs and 1,200 recent mothers recruited from the catchment areas of the surveyed ASHAs.

This multi-method approach provides a strong empirical foundation that includes detailed descriptions of health behaviors, community rituals, and beliefs, as well as insights into how various influencers and cultural values shape perinatal health decisions in rural Bihar.

**Appendix II. Qualitative Findings (FGDs and KIIs)**

**A. From Focus Group Discussions (FGDs) with Young Mothers, and Mother-in-Laws (Old mothers)**

**1. Influence on Perinatal Healthcare Decisions**

**1a. Temporal Domains of Influence**

**Pregnancy Period**

- **ASHAs** primarily influence institutional aspects:
  - Regular antenatal check-ups
  - Nutrition and supplementation
  - Registration for government schemes

Supporting quote: *"Medicine should not be consumed without consultation of doctor"* (MOTHER-FGD-2)

- **Dais** influence traditional protective practices:
  - Dietary restrictions
  - Movement restrictions
  - Traditional remedies

Supporting quote: *"Women not consume food of her own choice... [because] newborn saliva will come out"* (MIL-FGD-20)

**Labor and Delivery**

- **Dais** serve as primary assessors:
  - Initial labor confirmation
  - Decision to transfer to facility

Supporting quotes:

- *"Dai is called when the labor starts... Dai checks the contradiction"* (MIL-FGD-1)
- *"Before going to hospital for delivery, DAI is called to confirm her labor pain"* (MOTHER-FGD-6)
- *"First consult Dai, then only take to the hospital"* (MIL-FGD-3)

**Postpartum Period**

- **Traditional Practices** dominate immediate postpartum care:
  - Physical recovery practices
  - Traditional massages
  - Dietary management

Supporting quotes:

- *"Carom's seed [is used]... to keep the woman and her milk warm after delivery"* (MIL-FGD-1)
- *"Newborn and mother is immediately bathed after returning from the hospital"* (MIL-FGD-13)

**1b. Spheres of Authority**

**Traditional Knowledge Domain (Dais)**

- Ritual practices
- Body manipulation techniques
- Traditional remedies
- Cultural practices

Supporting quote: *"In Jaundice for new-borns, incantation is done by a spiritual person"* (MIL-FGD-1)

**Complex Ritual Systems**

Supporting quote: *"If the child is born in 27th Nakshatra, a ritual in which water from 27 wells, wood of 27 trees, pot made of 27 bamboo trees etc. is used to worship the God. Seven types of fruits, sweets, is prepared. Father sees the child in the shadow of oil or ghee, child is bathed with water of 27 wells"* (MOTHER-FGD-19)

**2. Mechanisms of Influence**

**2a. Dais' Mechanisms of Influence**

**Social Legitimacy**

- Generational trust
- Community integration
- Cultural alignment
- Family endorsement

Supporting quote: *"First consult Dai, then only take to the hospital"* (MIL-FGD-3), showing the primary position of trust held by Dais.

**Experiential Authority**

- Hands-on care delivery
- Immediate availability
- Physical techniques
- Crisis management

Supporting quote: *"Dai checks the contradiction"* (MIL-FGD-1), demonstrating their role in assessment and decision-making.

**2b. ASHAs' Mechanisms of Influence**

**Institutional Power**

- Government backing
- Scheme incentives
- Documentation authority
- Medical system linkages

Supporting quote: *"Consult doctor for any medicine"* (MOTHER-FGD-3), showing the integration with formal medical systems.

**3. Community Navigation Between Competing Recommendations**

**3a. Integration Patterns**

**Sequential Utilization**

- Initial traditional consultation
- Subsequent institutional care
- Post-institutional traditional care

Supporting quotes:

- *"First consult Dai, then only take to the hospital"* (MIL-FGD-3)
- *"Newborn and mother is immediately bathed after returning from the hospital"* (MIL-FGD-13)

**Traditional Preventive Practices**

Supporting quote: *"A scorpion is pasted on the wall with cow dung and after delivery dung cake is burnt on fire and warm compress is given to the child... [because] Child will never suffer from the poison of Scorpion"* (MIL-FGD-20)

**3b. Decision-Making Factors**

**Risk Assessment**

- Severity of condition
- Previous experiences
- Family history
- Community precedents

Supporting quote: *"Medicine should not be consumed without consultation of doctor"* (MOTHER-FGD-2), showing recognition of medical authority in certain contexts.

**B. From Key In-Depth Interviews (KIIs) with various community influencers, including ASHA and Dai**

**1. Divergent Approaches to Perinatal Care**

**Knowledge Systems and Practice Foundations**

ASHAs and Dais operate from fundamentally different knowledge systems that shape their approach to perinatal care. ASHAs work within the framework of biomedical knowledge, having received formal training through government health programs. Their recommendations are typically aligned with modern medical guidelines and public health protocols. As illustrated by *one ASHA's statement:*

*"We always advise regular ANC visits, TT vaccination, BP monitoring, and timely ultrasounds. These check-ups help us identify any complications early and ensure safe delivery."*

In contrast, Dais draw from generational wisdom and experiential knowledge passed down through traditional apprenticeships. Their practice incorporates cultural beliefs and traditional remedies, as evidenced by this Dai's perspective:

*"We have been helping mothers deliver babies for generations. We know which herbs help with morning sickness and which positions make delivery easier. These practices have worked for our mothers and grandmothers."*

**Integration of Traditional and Modern Practices**

Interestingly, both groups show varying degrees of adaptation to each other's approaches. Many ASHAs acknowledge the value of certain traditional practices when they don't conflict with medical recommendations:

*"Some traditional herbs for nausea are fine as long as they don't replace the prescribed medications. We need to respect these practices while ensuring mother's safety."*

Dais, while primarily rooted in traditional methods, increasingly recognize the importance of medical interventions in complicated cases:

*"When we see signs of danger like excessive bleeding or breech position, we now tell families to go to the hospital. Times have changed, and some complications need doctor's care."*

**2. Mechanisms of Influence**

**Community Trust and Authority**

Both ASHAs and Dais exercise influence through different forms of authority. ASHAs derive their authority from their official position and connection to the healthcare system:

*"People trust us because we can coordinate with the PHC (Primary Health Centre) and arrange ambulances in emergencies. We maintain proper records and ensure families get their government benefits."*

Dais, however, command respect through their deep community roots and long-standing relationships:

*"Families know us personally. We have helped deliver their other children, their relatives' children. They trust our experience and understanding of local customs."*

**Communication Strategies**

The analysis reveals distinct communication approaches. ASHAs typically employ structured health education methods:

*"We use flipcharts and mobile apps to explain the importance of iron tablets and proper nutrition. We organize group meetings where women can learn and ask questions."*

Dais often rely on informal, personalized communication:

*"We visit homes regularly, talk with the whole family, including mothers-in-law. We understand their fears and beliefs, and explain things in ways they understand."*

**3. Community Navigation Between Competing Recommendations**

**Decision-Making Dynamics**

Communities often find themselves navigating between traditional and modern healthcare recommendations. The analysis reveals several patterns in how families make these choices:

1. Severity-based selection: For routine care and minor issues, families often prefer traditional practices recommended by Dais. However, for complications or high-risk situations, they tend to follow ASHA's advice for medical intervention.
2. Complementary usage: Many families adopt a hybrid approach, following both ASHA and Dai recommendations when they don't conflict. As one ASHA noted:

*"Some women take their iron tablets but also drink the traditional herb tea. As long as it's safe, we don't object."*

**Conflict Resolution**

When ASHA and Dai recommendations conflict, several factors influence the resolution:

1. Family dynamics: The mother-in-law's preference often plays a crucial role in deciding whose advice to follow.
2. Previous experiences: Families' past experiences with both traditional and modern care influence their current choices.
3. Cost and accessibility: Practical considerations often determine whether families follow ASHA's recommendations for institutional delivery or opt for home delivery with a Dai.

**C. Comparative Analysis of ASHAs and Dais in Rural Bihar**

**1. Differential Influence on Perinatal Healthcare Decisions**

**Traditional Knowledge vs. Biomedical Approach**

The data reveals a fascinating contrast in how ASHAs and Dais approach perinatal care. Dais primarily operate through traditional knowledge systems, as evidenced by their practices. For instance, one Dai described their role as including traditional postpartum practices:

*"Massage the child and mother with oil and warm with smoke of carom seeds, bath mother with dettol after returning from hospital... suggestion to mother to avoid harmful food like peagon pea, brinjal, cauliflower, fava beans."*

This traditional approach is deeply rooted in local cultural practices and generational wisdom. In contrast, ASHAs represent a more formalized, biomedical approach to maternal healthcare, focusing on institutional delivery and modern healthcare practices.

**Temporal Scope of Care**

A notable difference emerges in the temporal scope of care provided by these two groups. Dais appear to have a more intensive involvement during the immediate perinatal period, particularly in the postpartum phase. As one Dai describes:

*"Give massage to the mother and child till 6 days... bath the child on CHHATHI... give massage to child till 6 month"*

**2. Mechanisms of Influence**

**Direct Physical Care vs. Health System Navigation**

Dais exercise their influence primarily through direct physical care and hands-on support. Their roles include:

- Physical examination: *"Check the delivery timing, adjust the position of child in womb"*
- Postpartum care: *"Clean and wash the dirty cloths till 6 days after delivery"*
- Traditional healing practices: *"Applied blue medicine on cord of child in two times in a day"*

**Adaptation to Changing Healthcare Landscape**

An interesting finding is how both groups are adapting to the increasing emphasis on institutional delivery. One Dai noted:

*"In the earlier time also support in home delivery but people don't call these days due to institutional delivery"*

This suggests a shift in roles, with Dais increasingly acting as complementary care providers rather than primary birth attendants.

**3. Community Navigation Between Competing Recommendations**

**Integration of Traditional and Modern Practices**

The data suggests an emerging pattern of integration rather than strict competition between ASHA and Dai recommendations. Dais appear to be incorporating modern healthcare advice into their traditional practice:

"Suggest to consult the doctor during pregnancy, Accompanies in Institutional [delivery]"

**Complementary Roles**

Rather than competing, the roles appear to be evolving into complementary functions:

- Dais focus on physical comfort and traditional postpartum practices
- ASHAs facilitate access to institutional healthcare and preventive care

**4. Emerging Themes**

**Role Evolution**

A significant theme is the evolution of Dais' roles in response to healthcare modernization. While maintaining their traditional expertise, they are adapting to support institutional delivery systems:

*"Support in delivery, give massage, cut cord stump, advice not to lift heavy weight, not climb stair, consume milk, fruits"*

**Cultural Bridging**

Both ASHAs and Dais serve as cultural bridges, though in different ways:

- Dais bridge traditional practices with modern healthcare needs
- ASHAs bridge institutional healthcare systems with community needs

**Continuity of Care**

Dais provide intensive, continuous care during the immediate postpartum period, which complements the more structured, milestone-based care provided by ASHAs.

**Appendix III. Focus Ethnography Findings**

**A. Familiarization and Overall Context**

Our field notes span multiple sites in **Samastipur District, Bihar** (Warisnagar, Morwa, Patori Blocks, etc.). We observed and interviewed:

- **Accredited Social Health Activists (ASHAs)** – coded as SMA01, SMA02, etc.
- **Dais** (traditional birth attendants) – typically older community women who assist with childbirth and postpartum care.
- **Other key public health workers**: ASHA trainers/facilitators (SMF, SMT), Mamtas (SMM), Auxiliary Nurse Midwives (ANMs), Medical Officer In-Charge (MOIC), Block Health Manager (BHM), Block Community Mobilizer (BCM), and staff from CARE.
- **Pregnant women, postpartum mothers, and their families** (mothers-in-law, husbands, siblings, in-laws).

The field notes illustrate real-time activities—**institutional deliveries**, **sterilization camps** (family planning days), **home visits for newborn care**, and everyday negotiations with families. References to dais emerge mostly in contexts of **home-based deliveries** or postpartum rituals.

**B. Major Themes and Sub-Themes**

**1. Contrasting Roles and Identities of ASHAs vs. Dais**

**1a. Position in the Community**

In our field data, ASHAs emerged as semi-formal, government-affiliated workers who are recognized both for their ability to **link** the community with public health systems and for the **incentives** they help secure. Dais, in contrast, typically hold a more **traditional** status, rooted in generational knowledge and personal relationships.

**Field Quote (ASHAs’ Semi-Formal Authority):**
*“She [SMA01] says that women are smarter these days and they do not need a lot of convincing now, as she has seen in her 12 years of working in the community. She has to do fewer home visits because women’s awareness levels have been raised.”*
*(Family Planning Day with SMA01, 15/02/19)*

**Field Quote (Dais as Embedded Elders):**
*“The dai refers to an incident from when her own mother used to work as a dai. She says that she learned by watching her mother... She did not have any formal training but they managed to deliver many babies at home.”*
*(Interview with Dai, 27/03/19)*

These excerpts highlight how ASHAs are viewed as **professionalizing** healthcare outreach, while Dais remain **integrated** into village life through personal connections. SMT01 (an ASHA trainer) further underscores this transformation:

*“Earlier ASHAs were not known in the village, but now every house knows them by name. Villagers recognize them as ‘someone’s daughter-in-law’ or as a ‘government worker.’”*
*(Interview with SMT01, 24/02/19)*

**1b. Training and Knowledge Sources**

- **ASHAs** rely on **formal training** modules (5, 6, 7...), government guidelines, and tools like **Mobile Kunji**.
- **Dais** typically draw on **experiential knowledge** passed down through older relatives.

**Field Quote (ASHAs and Mobile Kunji):**
*“SMA01 says that they use the ‘Mobile Kunji’ when working with pregnant mothers. It has information about the benefits of breastfeeding and diseases like diarrhea. She also says that now, with Mobile Kunji, they ‘have to talk less.’”*
*(Family Planning Day with SMA01, 15/02/19)*

**Field Quote (Dai’s Experiential Learning):**
*“The dai says that she has never received any formal training for what she does. If the woman is in labor already, she tells the family to call for a doctor. She wipes the child clean and cleans the area. She visits the child and the mother twice a day for six days after delivery.”*
*(Interview with Dai, 27/03/19)*

This contrast underscores how **institutionalized guidelines** shape the ASHA’s script, while Dais maintain a **culturally embedded** approach to birthing and postpartum practices.

**2. Mechanisms of Influence**

**2a. Persuasion, Trust, and Emotional Appeals**

Both ASHAs and Dais gain influence through different forms of **persuasion**.

- **ASHAs** emphasize government incentives (JSY, sterilization payouts) and assure hospital-based safety.
- **Dais** appeal to social and spiritual norms, commanding trust through longevity in the community.

**Field Quote (Monetary Incentives via ASHAs):**
*“Female sterilization surgery... is a stable method with an incentive of INR 2000 for the patient and INR 300 for the ASHA. Within 6 days of delivery, they are paid INR 3000 while the ASHA receives INR 400.”*
*(Family Planning Day with SMA01, 15/02/19)*

**Field Quote (Cultural/Spiritual Framing by Dais):**
*“She [dai] says if the child is bathed too early, it might ‘anger the ancestors.’ If families skip certain rituals, they risk the child’s health. She insists that’s why she comes every day for six days to massage the mother and newborn.”*
*(Interview with Dai, 27/03/19)*

Furthermore, **trust** is reinforced by how accessible these health workers are:

*“If we have an emergency at home… they don’t go at all unless the ASHA accompanies them. They trust us and feel that as long as they have an ASHA they will receive all the benefits at the PHC.”*
*(HBNC with SMA05, 01/03/19)*

**2b. Formal vs. Informal Negotiation**

- **ASHAs** negotiate at the PHC, ensuring families receive priority for lab tests, safe post-op rooms, and timely paperwork.
- **Dais** rarely engage with hospital bureaucracy, instead focusing on **home-based** tasks like postpartum massages and checking dilation.

**Field Quote (ASHAs’ Negotiation at PHC):**
*“SMA01 stands next to her patients in the lab, adjusting their hand positions for the blood test. She also asks family members to reserve beds in the post-op room quickly, otherwise someone else might take the space.”*
*(Family Planning Day with SMA01, 15/02/19)*

**Field Quote (Dais’ Limited Formal Role):**
*“I (dai) examine the pregnant woman and the ASHA does her documentation. I’m not involved in the government forms or incentives. My work is to see the mother, do massages, and check if everything is okay.”*
*(Interview with Dai, 27/03/19)*

These differing capacities highlight how ASHAs often **bridge bureaucratic gaps** while Dais remain intimately involved in the **cultural dimension** of perinatal care.

**3. Tensions and Synergies Between ASHAs and Dais**

**3a. Competing Recommendations**

We frequently observed **conflicts** around diet, timing of first feed, and choice of delivery location:

- **ASHAs** advocate a more **biomedical** approach: regular diet, immediate breastfeeding, institutional delivery.
- **Dais** (and older relatives) champion **traditional** or ritual-based practices, often postponing solid diets until chhatti or waiting for a pandit’s approval to initiate breastfeeding.

**Field Quote (Dietary Restrictions vs. Biomedical Advice):**
*“The mother says she is on a diet of ‘milk and ginger halwa’ only and will not eat grains before the sixth day. The ASHA tells her to eat rice and pulses, or else she’ll weaken, but the mother refuses until chhatti.”*
*(HBNC with SMA04, 27/02/19)*

**Field Quote (Home vs. Institutional Delivery):**
*“SMA05 says that last month, most deliveries happened at home. In some cases, the woman delivered by the time the ambulance arrived; in others, they simply refused to go to the PHC. But many do not need much convincing these days, as they know hospital deliveries are ‘safer.’”*
*(HBNC with SMA05, 01/03/19)*

**Field Quote (First Feed Controversies):**
*“Dai’s husband recalls that in their time, the child was not fed mother’s milk until a pandit gave the auspicious time. Meanwhile, the child was often fed goat’s or cow’s milk mixed with honey.”*
*(Interview with Dai’s Family, 27/03/19)*

**3b. Collaborative Practices or Overlaps**

Despite conflicts, we also noted **points of synergy**:

- Some dais welcome ASHAs’ involvement for safe delivery or if a complication arises.
- ASHAs recognize dais’ **value** in postpartum massages and local acceptance.

**Field Quote (Collaborative Cases):**
*“Dai mentions that she reminds the ASHA to call the research team if a labor case comes up. She says they coexist: ‘After the doctor leaves, my work begins. ASHA does the official paper, I do the massaging and check the new mother’s condition.’”*
*(Interview with Dai, 27/03/19)*

**Field Quote (Shared Breastfeeding Emphasis):**
*“While ASHA urges early initiation, the dai also says breastfeeding is important for the baby. She just differs on when it should begin, sometimes preferring to wait until the pandit’s instructions.”*
*(HBNC visits, multiple references)*

This **coexistence** demonstrates that families can receive **blended messages**, ultimately selecting which they find most relevant or credible.

**4. Community Navigation and Decision-Making**

**4a. Familial Gatekeepers (Mother-in-Law, Husband, Extended Family)**

Women’s decisions are rarely autonomous; mothers-in-law and husbands often **mediate** or shape final choices about delivery location, postpartum rituals, or family planning.

**Field Quote (Mother-in-Law’s Influence):**
*“One woman says she can’t get any rest at her marital home. She comes to her mother’s house for the sterilization surgery because her marital side doesn’t support her and there’s no one to look after her.”*
*(Family Planning Day with SMA01, 15/02/19)*

**Field Quote (Husband as Logistical Enabler):**
*“SMA01’s husband registers her patients early in the morning at the PHC to secure an earlier slot in the operation queue. He also repairs her shoe when it breaks from running around the hospital.”*
*(Family Planning Day with SMA01, 15/02/19)*

**4b. Women’s Agency and Selective Adoption**

Many women **blend** or **select** advice—adopting institutional deliveries or immunizations while preserving postpartum confinement, specialized diets, and fire pot usage.

**Field Quote (Blending Both Sides):**
*“One mother says, ‘I’ll take the iron tablets but will also keep the fire pot near the child—just in case. I don’t want to anger my elders.’”*
*(HBNC observations, general)*

Some families, especially those with higher socioeconomic status or education, **reject** ASHA advice if they feel they already know enough:

**Field Quote (Rejecting ASHA Advice):**
*“They [the well-off family] told the ASHA, ‘We get info from YouTube; we don’t need your suggestions on breastfeeding.’ The ASHA felt hurt but said she’ll still help when it’s time for immunizations.”*
*(HBNC with SMA05, 01/03/19)*

**5. Outcomes and Shifts in Perinatal Behaviors**

**5a. Adoption of Biomedical Care**

Over time, we observed a **steady rise** in institutional deliveries, influenced by:

1. **Safety**: fear of complications like postpartum hemorrhage.
2. **Financial incentives**: JSY scheme, free medication, etc.
3. **Persistent ASHA advocacy** in the field.

**Field Quote (Preference for PHC Deliveries):**
*“These days most people come to the PHC, though home births also happen sometimes. I had two of my children in a hospital and three at home before I became an ASHA.”*
*(Conversation with SMA02, 02/04/19)*

**Field Quote (Sterilization Focus):**
*“On Family Planning Day, the PHC is crowded with women who have come for tubal ligations. They often bring two or three family members with them, plus their ASHA.”*
*(Family Planning Day, multiple references)*

**5b. Persistence of Traditional Practices**

Even as biomedical care expands, families continue to honor **traditional norms** around postpartum confinement, dietary limitations, and rituals like chhatti.

**Field Quote (Postpartum Confinement):**
*“We observed a mother lying in a cordoned-off space (sori ghar). A fire pot was lit, and she used a makeshift toilet pit in the same room. The family said she would remain there until the sixth day’s ceremony.”*
*(Interview with postpartum family, 01/04/19)*

**Field Quote (Pandit Consultations):**
*“The father says he visited a pandit who told him that if the child was born after 1.15 PM, it would be ‘sattayisa.’ Since he was born in the morning, it’s considered good. He shares this news with the ASHA, who listens but doesn’t comment.”*
*(Interview with postpartum family, 01/04/19)*

Such practices reveal how cultural beliefs and **spiritual** interpretations remain integral to postpartum care, shaping decisions around bathing, feeding, and maternal confinement.

**C. Addressing Our Research Questions Directly**

**RQ1: How do ASHAs and Dais influence perinatal healthcare decisions differently?**

- ASHAs leverage **biomedical credentials**, incentives, and formal linkages to the PHC.
- Dais rely on **cultural embeddedness**, daily proximity, and spiritual reasoning.

**RQ2: Through what mechanisms do they exercise their influence?**

- **ASHAs**: Government guidelines, money-based persuasion (JSY/sterilization), and help navigating hospital bureaucracy.
- **Dais**: Emotional reassurance, ritual knowledge, generational experience, and trust built over years.

**RQ3: How do communities navigate between sometimes competing recommendations?**

- Families often **blend both** biomedical advice (institutional deliveries, immunizations) and local traditions (chhatti, fire pot, dietary restrictions).
- **Household gatekeepers** (mothers-in-law, husbands) may tip the balance.
- **Individual agency** appears in how some women selectively adopt or reject certain practices (e.g., using iron supplements yet maintaining postpartum rituals).

**Appendix IV. Quantitative Findings**

| Behavior | Rec response | Count | Percent |
| --- | --- | --- | --- |
| **Biomedical Behaviors** | | | |
| 4+ Antenatal Checkups | yes | 563 | 48.0 |
| Fast While Pregnant | no | 704 | 58.7 |
| Work While Pregnant | no | 426 | 35.5 |
| Dry Cord Care | no | 697 | 58.1 |
| Delayed Bathing (child) | no | 384 | 32.0 |
| Hospital Delivery | yes | 991 | 82.6 |
| Timely Initiation of Breastfeeding (TIBF) | yes | 784 | 65.3 |
| Feed Colostrum (child) | yes | 989 | 82.4 |
| Timely Registration (Preg) | yes | 619 | 51.6 |
| Avoid Cereal (postpart) | no | 362 | 30.2 |
| Iron (IFA) consumption | yes | 408 | 34.0 |
| Conceal Pregnancy (1^st^ Tri) | no | 1040 | 86.7 |
| **Neutral Behaviors** | | | |
| Dai Visit (pregnancy) | yes | 405 | 34.6 |
| Abstain Sex (pregnancy) | yes | 269 | 23.0 |
| Avoid Market (3 Tri) | yes | 724 | 61.8 |
| Consult Priest (pregnancy) | yes | 175 | 14.9 |
| Dai Visit (labor) | yes | 643 | 54.9 |
| Dai Visit (postpartum) | yes | 919 | 78.4 |
| Chhathi (celebration) | yes | 868 | 74.1 |
| Mom-NB Isolation | yes | 945 | 80.6 |

SMT 1. Counts and percentages of each sample responding ‘yes’ to the health behaviors analyzed in this study (N=1166).

SMT 2. Results of the selected logistic regression model that includes controls, influencers, behaviors, and influencer x behavior interactions

|  | OR | 2.5 % | 97.5 % | Estimate | Std. Error | z value | Pr(>\|z\|) |
| --- | --- | --- | --- | --- | --- | --- | --- |
| (Intercept) | 0.432 | 0.352 | 0.529 | -0.840 | 0.104 | -8.050 | 0.000 |
| ageclass21-24 | 0.995 | 0.875 | 1.132 | -0.005 | 0.066 | -0.072 | 0.942 |
| ageclass25-28 | 1.031 | 0.875 | 1.216 | 0.031 | 0.084 | 0.366 | 0.714 |
| ageclass29-33 | 0.904 | 0.733 | 1.115 | -0.101 | 0.107 | -0.944 | 0.345 |
| ageclass34+ | 1.051 | 0.800 | 1.380 | 0.049 | 0.139 | 0.355 | 0.722 |
| ageclass_married15-17 | 1.031 | 0.902 | 1.178 | 0.030 | 0.068 | 0.444 | 0.657 |
| ageclass_married18-20 | 1.084 | 0.938 | 1.254 | 0.081 | 0.074 | 1.094 | 0.274 |
| ageclass_married21+ | 1.139 | 0.875 | 1.484 | 0.130 | 0.135 | 0.967 | 0.334 |
| nkidscat2 | 0.874 | 0.767 | 0.996 | -0.134 | 0.067 | -2.015 | 0.044 |
| nkidscat3 | 0.810 | 0.692 | 0.947 | -0.211 | 0.080 | -2.644 | 0.008 |
| nkidscat4 | 0.796 | 0.657 | 0.964 | -0.228 | 0.098 | -2.335 | 0.020 |
| nkidscat5+ | 0.774 | 0.617 | 0.969 | -0.257 | 0.115 | -2.231 | 0.026 |
| educat1to7 | 0.941 | 0.819 | 1.082 | -0.060 | 0.071 | -0.849 | 0.396 |
| educat8to10 | 1.080 | 0.959 | 1.217 | 0.077 | 0.061 | 1.264 | 0.206 |
| educat11to13 | 1.196 | 0.995 | 1.439 | 0.179 | 0.094 | 1.900 | 0.057 |
| educat14to17 | 1.550 | 1.236 | 1.946 | 0.438 | 0.116 | 3.781 | 0.000 |
| wealthq2 | 1.105 | 0.966 | 1.264 | 0.100 | 0.068 | 1.462 | 0.144 |
| wealthq3 | 0.921 | 0.803 | 1.055 | -0.083 | 0.070 | -1.187 | 0.235 |
| wealthq4 | 0.998 | 0.866 | 1.151 | -0.002 | 0.073 | -0.026 | 0.979 |
| wealthq5 | 1.062 | 0.908 | 1.241 | 0.060 | 0.080 | 0.753 | 0.451 |
| Qancreg | 0.533 | 0.422 | 0.671 | -0.629 | 0.118 | -5.326 | 0.000 |
| Qcerealav | 4.310 | 3.193 | 5.847 | 1.461 | 0.154 | 9.474 | 0.000 |
| Qcolost | 4.006 | 2.996 | 5.380 | 1.388 | 0.149 | 9.302 | 0.000 |
| Qcordst | 2.034 | 1.536 | 2.693 | 0.710 | 0.143 | 4.957 | 0.000 |
| Qfast | 2.150 | 1.735 | 2.666 | 0.766 | 0.110 | 6.987 | 0.000 |
| Qhidep | 0.348 | 0.264 | 0.455 | -1.056 | 0.139 | -7.614 | 0.000 |
| Qifa | 0.122 | 0.087 | 0.167 | -2.104 | 0.164 | -12.795 | 0.000 |
| Qtibf | 0.474 | 0.352 | 0.634 | -0.746 | 0.150 | -4.967 | 0.000 |
| Qwork | 1.444 | 1.187 | 1.757 | 0.367 | 0.100 | 3.672 | 0.000 |
| Ifam | 3.030 | 2.666 | 3.447 | 1.109 | 0.066 | 16.906 | 0.000 |
| Ianm | 8.284 | 5.932 | 11.869 | 2.114 | 0.176 | 11.980 | 0.000 |
| Iasha | 10.169 | 8.204 | 12.699 | 2.319 | 0.111 | 20.826 | 0.000 |
| Idai | 0.864 | 0.642 | 1.162 | -0.146 | 0.151 | -0.968 | 0.333 |
| Ifriendrelnei | 1.852 | 1.538 | 2.234 | 0.616 | 0.095 | 6.475 | 0.000 |
| Iprivclinic | 2.168 | 1.597 | 2.970 | 0.774 | 0.158 | 4.893 | 0.000 |
| Igovdoc | 13.560 | 8.415 | 22.806 | 2.607 | 0.254 | 10.284 | 0.000 |
| Irmp | 1.886 | 0.969 | 3.785 | 0.634 | 0.347 | 1.831 | 0.067 |
| Imedia | 3.607 | 1.687 | 8.266 | 1.283 | 0.404 | 3.177 | 0.001 |
| Itraining | 4.846 | 1.540 | 18.617 | 1.578 | 0.622 | 2.536 | 0.011 |
| IOTHER | 1.724 | 1.040 | 2.894 | 0.545 | 0.260 | 2.092 | 0.036 |
| Qfast:Ifam | 0.256 | 0.194 | 0.339 | -1.361 | 0.143 | -9.530 | 0.000 |
| Qcordst:Ifam | 0.101 | 0.071 | 0.142 | -2.293 | 0.176 | -12.996 | 0.000 |
| Qhidep:Ifam | 0.256 | 0.175 | 0.373 | -1.362 | 0.193 | -7.049 | 0.000 |
| Qtibf:Ifam | 1.131 | 0.758 | 1.701 | 0.123 | 0.206 | 0.599 | 0.549 |
| Qcolost:Ifam | 0.281 | 0.196 | 0.403 | -1.270 | 0.184 | -6.883 | 0.000 |
| Qcerealav:Ifam | 0.374 | 0.271 | 0.515 | -0.984 | 0.164 | -6.001 | 0.000 |
| Qwork:Ianm | 0.169 | 0.071 | 0.431 | -1.777 | 0.458 | -3.881 | 0.000 |
| Qcordst:Ianm | 0.444 | 0.269 | 0.728 | -0.811 | 0.254 | -3.198 | 0.001 |
| Qhidep:Ianm | 0.053 | 0.017 | 0.134 | -2.943 | 0.514 | -5.723 | 0.000 |
| Qifa:Ianm | 1.258 | 0.719 | 2.195 | 0.229 | 0.284 | 0.807 | 0.420 |
| Qtibf:Ianm | 3.216 | 1.752 | 6.030 | 1.168 | 0.314 | 3.716 | 0.000 |
| Qancreg:Ianm | 0.254 | 0.146 | 0.441 | -1.370 | 0.281 | -4.867 | 0.000 |
| Qcerealav:Ianm | 0.199 | 0.102 | 0.398 | -1.615 | 0.345 | -4.682 | 0.000 |
| Qfast:Iasha | 0.812 | 0.319 | 2.508 | -0.208 | 0.516 | -0.404 | 0.686 |
| Qwork:Iasha | 0.455 | 0.255 | 0.846 | -0.787 | 0.304 | -2.585 | 0.010 |
| Qcordst:Iasha | 0.268 | 0.181 | 0.395 | -1.318 | 0.198 | -6.644 | 0.000 |
| Qhidep:Iasha | 0.203 | 0.125 | 0.325 | -1.593 | 0.243 | -6.557 | 0.000 |
| Qifa:Iasha | 1.710 | 1.096 | 2.693 | 0.536 | 0.229 | 2.341 | 0.019 |
| Qtibf:Iasha | 2.618 | 1.524 | 4.685 | 0.962 | 0.285 | 3.373 | 0.001 |
| Qcolost:Iasha | 0.848 | 0.492 | 1.541 | -0.164 | 0.290 | -0.568 | 0.570 |
| Qancreg:Iasha | 0.940 | 0.658 | 1.343 | -0.062 | 0.182 | -0.343 | 0.732 |
| Qcerealav:Iasha | 0.358 | 0.206 | 0.650 | -1.027 | 0.291 | -3.525 | 0.000 |
| Qcordst:Idai | 0.344 | 0.173 | 0.652 | -1.068 | 0.337 | -3.173 | 0.002 |
| Qtibf:Idai | 8.659 | 4.247 | 18.327 | 2.159 | 0.371 | 5.811 | 0.000 |
| Qcolost:Idai | 1.608 | 0.851 | 3.123 | 0.475 | 0.331 | 1.437 | 0.151 |
| Qcerealav:Idai | 0.263 | 0.153 | 0.449 | -1.334 | 0.274 | -4.863 | 0.000 |
| Qfast:Ifriendrelnei | 0.216 | 0.151 | 0.305 | -1.534 | 0.179 | -8.578 | 0.000 |
| Qwork:Ifriendrelnei | 0.751 | 0.520 | 1.088 | -0.287 | 0.188 | -1.522 | 0.128 |
| Qcordst:Ifriendrelnei | 0.189 | 0.113 | 0.309 | -1.667 | 0.257 | -6.487 | 0.000 |
| Qhidep:Ifriendrelnei | 0.577 | 0.353 | 0.921 | -0.550 | 0.244 | -2.254 | 0.024 |
| Qcolost:Ifriendrelnei | 0.603 | 0.384 | 0.958 | -0.505 | 0.233 | -2.168 | 0.030 |
| Qancreg:Ifriendrelnei | 1.310 | 0.864 | 2.002 | 0.270 | 0.214 | 1.263 | 0.207 |
| Qcerealav:Ifriendrelnei | 0.685 | 0.482 | 0.977 | -0.378 | 0.180 | -2.101 | 0.036 |
| Qwork:Iprivclinic | 12.570 | 4.889 | 42.833 | 2.531 | 0.540 | 4.690 | 0.000 |
| Qcordst:Iprivclinic | 0.135 | 0.075 | 0.238 | -2.004 | 0.294 | -6.810 | 0.000 |
| Qifa:Iprivclinic | 11.558 | 6.242 | 21.752 | 2.447 | 0.318 | 7.696 | 0.000 |
| Qtibf:Iprivclinic | 0.393 | 0.176 | 0.853 | -0.933 | 0.401 | -2.325 | 0.020 |
| Qcolost:Iprivclinic | 0.639 | 0.301 | 1.439 | -0.449 | 0.396 | -1.132 | 0.258 |
| Qancreg:Iprivclinic | 0.814 | 0.154 | 4.099 | -0.205 | 0.814 | -0.252 | 0.801 |
| Qcordst:Igovdoc | 0.126 | 0.063 | 0.251 | -2.070 | 0.354 | -5.849 | 0.000 |
| Qcolost:Igovdoc | 0.271 | 0.099 | 0.881 | -1.304 | 0.549 | -2.376 | 0.017 |
| Qcerealav:Igovdoc | 0.089 | 0.037 | 0.225 | -2.416 | 0.458 | -5.278 | 0.000 |
| Qcordst:Irmp | 0.040 | 0.010 | 0.126 | -3.220 | 0.632 | -5.095 | 0.000 |
| Qifa:Imedia | 0.185 | 0.035 | 1.223 | -1.685 | 0.891 | -1.890 | 0.059 |
| Qtibf:IOTHER | 1.448 | 0.388 | 5.315 | 0.371 | 0.662 | 0.560 | 0.576 |
| Qcolost:IOTHER | 1.773 | 0.427 | 12.196 | 0.573 | 0.816 | 0.702 | 0.483 |
| Qancreg:IOTHER | 1.565 | 0.367 | 7.304 | 0.448 | 0.749 | 0.598 | 0.550 |

SMT 4. Results of moderation analysis that includes controls and interaction between each moderator (wealth, caste, and religion) and ASHA interaction score.

|  | Wealth | Caste | Religion |
| --- | --- | --- | --- |
| (Intercept) | 1.891*** | 1.888*** | 1.927*** |
|  | [1.846, 1.937] | [1.840, 1.936] | [1.862, 1.991] |
| Parity2 | -0.075** | -0.073** | -0.077** |
|  | [-0.124, -0.025] | [-0.123, -0.023] | [-0.127, -0.027] |
| Parity3 | -0.120*** | -0.124*** | -0.127*** |
|  | [-0.180, -0.061] | [-0.183, -0.065] | [-0.187, -0.068] |
| Parity4 | -0.108** | -0.114** | -0.117** |
|  | [-0.182, -0.034] | [-0.187, -0.041] | [-0.190, -0.044] |
| Parity5+ | -0.231*** | -0.240*** | -0.241*** |
|  | [-0.323, -0.140] | [-0.331, -0.148] | [-0.332, -0.149] |
| EDU1to7 | 0.029 | 0.030 | 0.036 |
|  | [-0.027, 0.085] | [-0.026, 0.086] | [-0.020, 0.091] |
| EDU8to10 | 0.085*** | 0.095*** | 0.096*** |
|  | [0.040, 0.131] | [0.050, 0.139] | [0.052, 0.140] |
| EDU11to13 | 0.145*** | 0.154*** | 0.161*** |
|  | [0.079, 0.211] | [0.091, 0.217] | [0.098, 0.224] |
| EDU14to17 | 0.213*** | 0.220*** | 0.227*** |
|  | [0.141, 0.286] | [0.151, 0.290] | [0.158, 0.297] |
| Age21-24 | 0.018 | 0.020 | 0.023 |
|  | [-0.032, 0.067] | [-0.029, 0.070] | [-0.027, 0.072] |
| Age25-28 | 0.055+ | 0.064* | 0.064* |
|  | [-0.008, 0.119] | [0.002, 0.127] | [0.001, 0.126] |
| Age29-33 | -0.007 | 0.002 | 0.002 |
|  | [-0.091, 0.077] | [-0.082, 0.086] | [-0.082, 0.086] |
| Age34+ | 0.052 | 0.063 | 0.060 |
|  | [-0.058, 0.162] | [-0.047, 0.173] | [-0.050, 0.170] |
| ASHA_Int_c | 0.021*** | 0.026*** | 0.014*** |
|  | [0.018, 0.023] | [0.021, 0.031] | [0.006, 0.022] |
| wealth_diff_c | -0.011 |  |  |
|  | [-0.025, 0.004] |  |  |
| ASHA_Int_c × wealth_diff_c | 0.002 |  |  |
|  | [-0.001, 0.004] |  |  |
| caste_diff1 |  | -0.002 |  |
|  |  | [-0.037, 0.033] |  |
| ASHA_Int_c × caste_diff1 |  | -0.008** |  |
|  |  | [-0.014, -0.002] |  |
| relig_diff1 |  |  | -0.047+ |
|  |  |  | [-0.101, 0.006] |
| ASHA_Int_c × relig_diff1 |  |  | 0.007 |
|  |  |  | [-0.002, 0.016] |
| Num.Obs. | 1158 | 1158 | 1158 |
| AIC | 4830.6 | 4827.8 | 4830.0 |
| BIC | 4916.5 | 4913.7 | 4915.9 |
| Log.Lik. | -2398.304 | -2396.886 | -2397.978 |
